# sEMG-Based Hand Posture Recognition Considering Electrode Shift, Feature Vectors, and Posture Groups

**DOI:** 10.3390/s21227681

**Published:** 2021-11-18

**Authors:** Jongman Kim, Bummo Koo, Yejin Nam, Youngho Kim

**Affiliations:** Department of Biomedical Engineering, Yonsei University, Wonju 26493, Korea; jmkim0127@ybrl.yonsei.ac.kr (J.K.); bmk726@ybrl.yonsei.ac.kr (B.K.); namyj_1007@naver.com (Y.N.)

**Keywords:** surface electromyography, pattern recognition, artificial neural network, electrode shift, hand posture, feature vector, human-computer interaction, armband sensor

## Abstract

Surface electromyography (sEMG)-based gesture recognition systems provide the intuitive and accurate recognition of various gestures in human-computer interaction. In this study, an sEMG-based hand posture recognition algorithm was developed, considering three main problems: electrode shift, feature vectors, and posture groups. The sEMG signal was measured using an armband sensor with the electrode shift. An artificial neural network classifier was trained using 21 feature vectors for seven different posture groups. The inter-session and inter-feature Pearson correlation coefficients (PCCs) were calculated. The results indicate that the classification performance improved with the number of training sessions of the electrode shift. The number of sessions necessary for efficient training was four, and the feature vectors with a high inter-session PCC (*r* > 0.7) exhibited high classification accuracy. Similarities between postures in a posture group decreased the classification accuracy. Our results indicate that the classification accuracy could be improved with the addition of more electrode shift training sessions and that the PCC is useful for selecting the feature vector. Furthermore, hand posture selection was as important as feature vector selection. These findings will help in optimizing the sEMG-based pattern recognition algorithm more easily and quickly.

## 1. Introduction

Gestures, involving the physical movements of the hands, face, or body, is a form of communication used to convey meaningful information or interact with the environment [1]. Among the various gestures, those typically applied in machine learning algorithms as the interface of human-computer interaction (HCI) are hand gestures. This is because they constitute the most natural and efficient movements in daily life [2]. As an HCI interface, a hand gesture recognition system has three advantages [3]. The first advantage is the ease of hygiene management through a contactless interface. This contactless interface helps in maintaining hygienic conditions for the user by preventing contamination due to contact. Therefore, hand gesture recognition systems are useful in clinical applications such as healthcare systems. The second advantage is that a hand gesture recognition system can be applied as an alternative to overcome physical disabilities. It is easy to apply this system to an assistive device, such as a home-care system or an IoT system controller, for the disabled or elderly who have difficulty in moving. Furthermore, the need for a gesture-based HCI interface is increasing, owing to the increasing number of people who can only communicate through hand gestures (e.g., sign language for the deaf). The third advantage is that considerable data and commands can be easily managed by the intuitive movements.

Studies on hand gesture recognition have predominantly used one of the two following technologies: computer vision or wearable sensors. Computer vision-based systems use one or more cameras to recognize hand gestures. Shin et al. developed a six-hand gesture recognition system using a low-priced USB color camera and entropy analysis; they achieved a classification accuracy of 97.2% [4]. Stergiopoulou and Papamarkos were developed a neural network-based hand gesture recognition system using one camera and thirty-one classified hand gestures with an accuracy of 90.0% [5]. However, these systems encountered the problem of environmental factors (e.g., shadow, lighting, background, and camera position) affecting the classification performance. They exemplify the difficulty involved in optimizing computer vision-based recognition systems under environmental factors, varying with respect to time and place. Furthermore, the camera, which has a high resolution for accurate gesture recognition, is expensive and has low portability, and the output data file is very large.

Wearable sensor-based systems use non-invasive sensors on the user’s skin. The bio-signal or the motion of the user is detected and measured by the non-invasive sensors, and the measured data are used to recognize the gestures. A data glove is predominantly used in studies on wearable sensor-based hand gesture recognition, and is useful for measuring the posture and gesture of the hand accurately. Nam et al. classified ten hand gestures with an accuracy of 80.0% using the VLP data glove and hidden Markov model (HMM) [6]. Additionally, Yin et al. developed a hand gesture recognition system that recognizes nine hand gestures, with an accuracy of 99.8%, using the data glove and neural network algorithm [7]. However, it is difficult to use the data glove-based gesture recognition system in daily life, owing to the high price of the glove and consequent contamination from sweat and oil after long-term use. Furthermore, the data glove restricts the natural hand gestures of the user, and the glove design causes discomfort because of repetitive donning and doffing in daily life.

Recently, several studies have applied sEMG sensors to overcome problems encountered by hand gesture recognition systems. sEMG is a non-invasive method for measuring the fine bio-signal of muscle activation, and the sEMG signal contains extensive information about the activity of neurons from the spinal cord to the muscle fibers. Therefore, sEMG is widely used in clinic and rehabilitation and bio-signal-based control systems for HCI. Kim et al. classified four-hand gestures from one sEMG sensor and achieved a classification accuracy of approximately 94.0% using a combination of the K-nearest neighbor (KNN) and Bayes classifier [8]. Shi et al. developed a four-hand gesture recognition algorithm and achieved an accuracy of 94.0% using two sEMG sensors and a KNN classifier [9]. These previous studies suggested using each muscle belly for the positions of the sEMG sensors, to avoid crosstalk between sEMG signals; however, this decreases the practicality of using the gesture recognition system because finding the muscle belly is difficult for non-expert users in daily life.

A wearable sensor, which has the design of an armband or a wristband, has been suggested to increase the practicality for non-expert users in daily life. Jiang et al. developed a wristband-type sEMG sensor that includes four channel sEMG modules and one inertial measurement unit (IMU). They classified eight hand gestures with an accuracy of 92.6% using a linear discriminant analysis classifier [10]. Abreu et al. classified twenty static hand gestures with an accuracy of 98.6% using a support vector machine (SVM) classifier and a commercial sensor called the Myo Armband (Thalmic Labs, Kitchener, Canada) [11]. However, the two main problems—electrode shift and feature vector selection—remain unsolved.

Electrode shift is a common issue that arises during donning and doffing of a sensor in daily life. Many previous studies fixed the positions of both the sEMG and wearable sensors to avoid misclassifications that result from electrode shift. Feature vector selection is an important process in the development of a pattern recognition algorithm [12,13]. Previous studies analyzed the classification performance of each feature vector and applied the feature vector that delivered the best performance in the hand gesture recognition algorithm. However, various factors, such as the sensor performance, limb position, and electrode shift, easily affect the classification performance of the feature vector. Therefore, feature vector selection, based on classification performance, is inefficient in the development of the pattern recognition algorithm. In previous studies on efficient pattern recognition, a principal component analysis (PCA) and a genetic algorithm (GA), both of which reduce the dimension of the feature vector and minimize the data complexity, were suggested for feature vector selection [14,15]; however, few studies have been conducted on feature vector selection that consider the electrode shift.

Many previous studies developed hand gesture recognition systems, but the number of target gestures are limited because of the limitations of the classification performances of the algorithms and the efficiencies of the systems. Therefore, target gestures were selected with reference to previous studies. For the HCI interface, Wahid et al. developed a classification algorithm for the target gestures of a fist, wave in, and wave out [16], and Zhang et al. developed an algorithm for the target gestures of a double tap, wave in, wave out, fingers spread, and fist [17]. Castiblanco et al. selected twelve hand wrist gestures to develop a gesture recognition algorithm for stroke rehabilitation [18]. Kim et al. selected thirty-eight Korean finger languages in a finger language recognition study [19], and seven hand gestures were selected in a myoelectric hand prosthesis control study [20]. Andrad et al. developed a hand gesture recognition algorithm for the target gestures of a cylindrical grasp, tip pinch, and hook (snap, palmar pinch, spherical grasp, and lateral pinch) [21]; furthermore, they reported that the classification accuracy decreased with similarity in the same-gesture group (precision grasp: tip, palmar and lateral; power grasp: cylindrical, hook, and spherical). Therefore, these previous studies were performed to improve the classification performance with a selected target gesture. However, selected gestures could be replaced with other gestures which had a similar form or function. Therefore, the analysis about the gesture selection was important to improve the gesture recognition algorithm, but few studies have been conducted on classification performance in accordance with the gesture selection type.

In this study, a hand posture recognition algorithm was developed, taking into consideration the electrode shift, feature vectors, and posture types. A custom armband-type multi-channel sEMG sensor was used to measure the sEMG signal on the forearm. Experiments were conducted with electrode shifts and the measured sEMG signal was used to calculate twenty-one feature vectors. Twelve hand postures were selected after a literature review, and seven posture groups were formed considering the function and muscle activation of each hand posture. The classification accuracy and inter-session and inter-feature PCCs were analyzed for the PCC-based feature vector selection.

## 2. Materials and Methods

### 2.1. Participants

Ten healthy right-handed adults (7 males, 3 females, 24.1 ± 0.7 years) without neurological disorders were recruited to participate in this study. All participants were fully informed of any of the risks associated with the experiments, and they gave their written consent to participate in this study. The experimental procedure was approved by the Yonsei University Mirae Institutional Review Board (1041849-201704-BM-018-01).

### 2.2. Equipment

**Figure 1:**
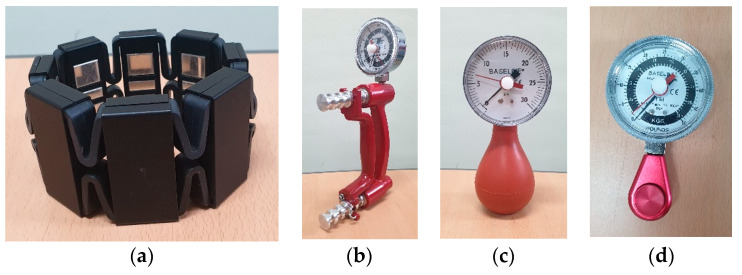
Armband-type multi-channel sEMG sensor (**a**) and hand dynamometers (**b**–**d**).

Figure 1 shows the armband-type sEMG sensor and Baseline hand dynamometers (Fabrication Enterprises, Inc., White Plains, NY, USA) used in this experiment. The custom armband-type eight-channel sEMG sensor was used to measure the sEMG signal with a sampling frequency of 500 Hz [19]. Each participant wore the armband sensor on their right forearm, and the main module of the armband sensor was placed on the belly area of the anterior part of the forearm during wrist flexion (around the flexor carpi radialis). The hand dynamometers were used to perform each hand posture with fixed grasp force.

### 2.3. Experimental Procedures

**Figure 2:**
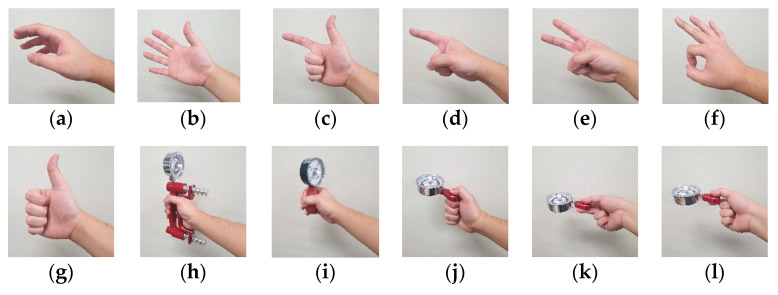
Twelve hand postures selected for use in this study: (**a**) rest, (**b**) spread, (**c**) scissor-sign, (**d**) finger pointing, (**e**) V-sign, (**f**) O.K.-sign, (**g**) thumb-up (hook), (**h**) cylindrical grasp, (**i**) spherical grasp, (**j**) lateral pinch, (**k**) palmar pinch, (**l**) tip pinch.

Twelve hand postures were selected after a literature review [10,21,22,23,24,25,26,27,28,29,30] (Figure 2); the function of each of these hand postures is presented in Table 1. 

In this study, seven hand posture groups were constructed, and the classification performances of each posture group were analyzed to find the most efficient hand posture group for the development of a hand posture recognition algorithm. The posture groups were determined using the function and activated muscles of each hand posture. Group 1 was composed of the most frequently used and important hand postures identified in previous studies. Group 2 and Group 5 consisted of the postures in Group 1 and the finger-pointing or scissor-sign postures, respectively, which are postures used to point to objects. Group 3 and Group 4 consisted of the postures in Group 1 and postures such as a tip pinch and spherical grasp, which have the same function as those included in Group 1. Seven different hand postures were selected to be part of Group 6, considering the overlap of the functions and activated muscles on each hand posture. Finally, Group 7 included all of the listed hand postures. 

In an sEMG-based gesture recognition algorithm, force and muscle fatigue were classed as critical factors to increase the variability of the sEMG signal. Therefore, all hand postures were performed with 20% maximum voluntary contraction (MVC) to avoid muscle fatigue and the confounding factor with the grasp force [20,31]. Hydraulic-hand and pneumatic-hand dynamometers, and hydraulic pinch gauge (Fabrication Enterprises, Inc., White Plains, NY, USA) were used for the grasp and pinch postures, respectively. All participants practiced maintaining 20% MVC in all hand postures except the rest posture. Postures without the use of the hand dynamometers were performed with the same force as displayed in the practice. The participants were seated on chairs and performed each hand posture for 5 s in a random order, and the experimental session was repeated 10 times. All participants took sufficient rests, removing the sensor between experimental sessions, and the sensor was worn again before the next session.

### 2.4. Feature Vector Extraction

The sEMG signal was filtered using the fourth bandpass filter with a bandwidth of 15–250 Hz, and the filtered sEMG signal was used to calculate the feature vectors. In this study, the feature vectors were selected in the time domain corresponding to real-time application [32]. Twenty-one time-domain feature vectors and their corresponding formulas are presented in Table 2. The hand posture recognition algorithm was applied to feature vectors of a single type. The feature vectors were calculated with a window size of 250 ms and a window shift of 10 ms, as recommended in a previous study [33]. The feature vectors of AR and CC were calculated using various orders of 1 to 10.

The threshold-based feature vectors, such as ZC, MYOP, WAMP, and SSC, were calculated using the predefined threshold values. In previous studies, the threshold value was selected from 50 µV to 100 mV considering the gain of the sEMG sensor and background noise. The optimization of the threshold value is crucial because a considerably low threshold value leads to the transmission of unwanted information from the background noise, and a considerably high threshold value misses important information for pattern recognition. However, finding the optimized threshold value requires significant amounts of time and high costs for the gesture recognition system. Therefore, many previous studies used threshold values from other studies, rather than optimizing the threshold values in their systems; moreover, few studies have been conducted to find the most suitable threshold value for each feature vector. Kamavuako et al. suggested Equation (1) for defining various threshold values using a factor *R* and the *RMS* value from the sEMG signal at rest [34]. The equation proved useful in reducing the time and cost for the optimization of the threshold values in the gesture recognition algorithm:

(1) $$Threshold\;value=R\times RMS_{sEMG^{at\;rest}},$$

Consequently, the equation was used in this study to optimize the threshold value for each feature vector, and a factor *R* was applied in increments of 0.5, from 0.0 to 10.0. In addition, the threshold values used in previous studies [13,20,35,36,37,38,39,40,41,42,43] were applied in the hand posture recognition algorithm.

### 2.5. Classifier

An artificial neural network (ANN) is a machine learning algorithm that was developed by simulating a biological neural network in the brain of a human or animal. The ANN was constructed with the input layer, hidden layer, and output layer, using artificial neurons, known as the node, which then classified the input signals through learning processes such as the backpropagation algorithm. The joint function between the input/hidden/output layers was easily estimated, and the classification was performed quickly.

In the input and the output layers, the number of nodes were determined by the feature vector and the target class. However, the hidden layer was able to change the number of nodes. The optimization of the hidden layer’s nodes was important because insufficient nodes caused underfitting, while excessive nodes caused overfitting [44,45]. In this study, 8 nodes in the input layer (1 sEMG feature × 8 channels) and 6–12 nodes in the output layer (the number of hand posture) were determined in ANN, and there were 17 nodes in the hidden layer according to a previous study [46]. Both the training and testing of the ANN classifier were performed through 10-fold cross validation using the MATLAB Deep Learning Toolbox (Mathworks, Inc., Natick, MA, USA).

### 2.6. Performance Evaluation

In this study, twenty-one time-domain feature vectors and two feature combinations (Hudgins’ set: MAV, WL, ZC, and SSC [38] and Du’s set: IEMG, VAR, WL, ZC, SSC, and WAMP [47]) from previous studies were applied to the ANN classifier. The classification accuracy was analyzed based on the number of training sessions and considering the electrode shift, and the PCC was calculated to analyze the linear relationship between the sessions or the feature vectors. Many previous studies suggested various methods to select the feature vectors for the improvement of the classification performance [10,12,14,15]. Correlation-based feature vector selection was primarily used to evaluate the way each feature vector is able to distinguish a gesture or verify the similarity of the information of feature vectors. In this study, the inter-session PCC was used to analyze the correlation between the sessions with the electrode shift, and the similarity of the feature vectors was analyzed by inter-feature PCC. A factor *r* represented the linear relationships as follows: weak linear relationship: 0 < *r* ≤ 0.3; moderate linear relationship: 0.3 < *r* ≤ 0.7; and strong linear relationship: 0.7 < *r* ≤ 1.0.

A statistical analysis was performed using IBM SPSS Statistics (IBM, Corp., Armonk, NY, USA), and the results of the evaluation were determined as nonparametric. The Kruskal–Wallis H test and pairwise comparison were performed to examine the differences in classification accuracy in accordance with the threshold values, feature vector orders, and the number of training sessions. The statistical significance was set at *p* < 0.05 for all tests.

## 3. Results

### 3.1. Classification Accuracy Based on Threshold Values and Feature Vector Orders

In this study, various threshold values obtained using Equation (1), and from previous studies, were applied to calculate the threshold-based feature vectors. The RMS value of the sEMG signal at rest was 3.3 mV, and the threshold values were defined in the range of 0.0–33.0 mV following accordance with Equation (1), with a step size of 1.65 mV. The range of the threshold values from Equation (1) included all threshold values from previous studies. 

**Figure 3:**
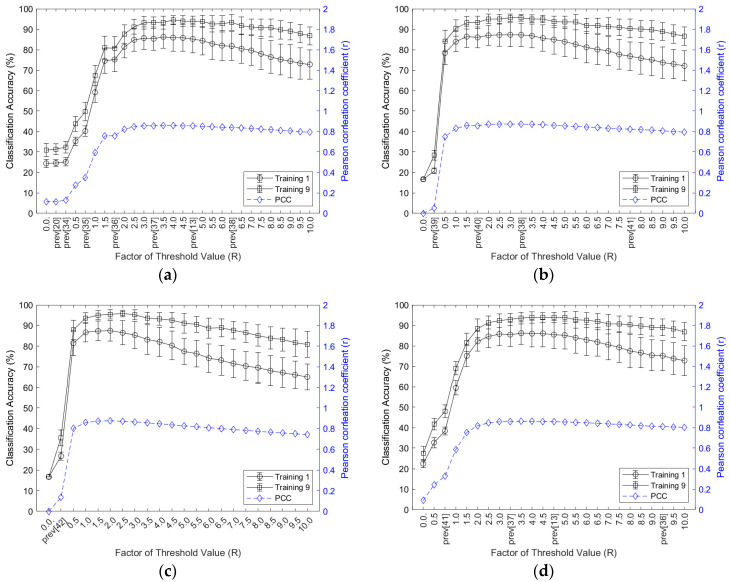
Classification accuracy and inter-session PCC of Group 1 based on threshold values: (**a**) ZC; (**b**) WAMP; (**c**) MYOP; (**d**) SSC; prev: threshold value from the previous study.

Figure 3 shows the classification accuracy and inter-session PCC of Group 1 in accordance with the threshold values (the results for the other groups are shown in Figures S1–S6). All threshold-based feature vectors showed improved classification accuracies corresponding with an increasing number of training sessions. The classification accuracy and inter-session PCC were dramatically improved by increasing the threshold value, and then maintained at the constant level. However, excessive threshold values were found to cause a degradation of the classification accuracy and inter-session PCC. The best threshold values depended on the feature vector.

The threshold value of 13.2 mV exhibited the best classification accuracy in ZC with nine training sessions (Group 1: 94.5 ± 2.1%, Group 2: 91.2 ± 2.3%, Group 3: 85.7 ± 4.3%, Group 4: 86.6 ± 4.6%, Group 5: 89.4 ± 2.9%, Group 6: 93.1 ± 3.8%, Group 7: 76.7 ± 4.7%). The best threshold value of WAMP with nine training sessions was 9.9 mV (Group 1: 95.6 ± 1.8%, Group 2: 92.7 ± 1.6%, Group 3: 86.1 ± 4.4%, Group 4: 89.5 ± 3.7%, Group 5: 90.8 ± 3.2%, Group 6: 94.5 ± 3.5%, Group 7: 79.3 ± 4.9%). In MYOP and SSC, with nine training sessions, the best threshold values were 6.6 mV (Group 1: 95.4 ± 2.2%, Group 2: 92.5 ± 2.2%, Group 3: 86.7 ± 4.7%, Group 4: 89.8 ± 3.8%, Group 5: 90.7 ± 3.0%, Group 6: 94.7 ± 3.1%, Group 7: 79.1 ± 4.7%) and 13.2 mV (Group 1: 93.9 ± 2.8%, Group 2: 91.1 ± 2.4%, Group 3: 84.6 ± 4.6%, Group 4: 86.9 ± 5.0%, Group 5: 88.9 ± 2.4%, Group 6: 92.9 ± 3.7%, Group 7: 76.6 ± 5.1%), respectively. Furthermore, the appropriate ranges of the threshold values were 8.25–23.1 mV, 6.6–18.15 mV, 3.3–14.85 mV, and 8.25–23.1 mV in the feature vectors of ZC, WAMP, MYOP, and SSC, respectively. These ranges of threshold values were similar for the posture group in each feature vector. The classification accuracy and inter-session PCC were high (*r* > 0.8) for the feature vectors with appropriate ranges of threshold values.

**Figure 4:**
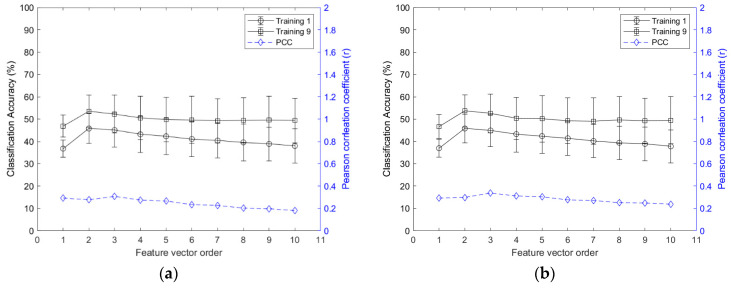
Classification accuracy and inter-session PCC of Group 1 based on the orders: (**a**) AR; (**b**) CC.

The classification accuracy and inter-session PCC of Group 1, for the feature vectors of AR and CC, are shown in Figure 4 (results of the other groups are presented in Figures S7–S12). The second order exhibited the best classification accuracies for both AR (Group 1: 53.6 ± 7.2%; Group 2: 49.1 ± 6.0%; Group 3: 47.6 ± 6.6%; Group 4: 49.1 ± 7.0%; Group 5: 48.9 ± 6.2%; Group 6: 52.2 ± 8.2%; Group 7: 35.9 ± 5.8%) and CC (Group 1: 53.7 ± 7.2%; Group 2: 49.5 ± 6.6%; Group 3: 46.8 ± 6.3%; Group 4: 49.6 ± 6.5%; Group 5: 49.2 ± 6.9%; Group 6: 52.2 ± 8.6%; Group 7: 36.5 ± 5.9%); however, there was no significant difference between the orders of AR and CC.

### 3.2. Classification Accuracy and Inter-Session PCC

Table 3 present the classification accuracies and inter-session PCCs according to the number of training sessions and feature vectors in Group 1, respectively (results of the other groups are presented in Tables S1–S6). The classification accuracy was improved with an increasing number of training sessions in all feature vectors, and a significant improvement was observed for four or more training sessions. Although the classification accuracies for five to nine training sessions (more than four) were higher than those for four training sessions, no significant difference was found. Across the four training sessions, feature vectors with high inter-session PCCs (*r* > 0.7; strong linear relationship) exhibited higher classification accuracies (Group 1: >90.0%, Group 2: >88.0%, Group 3: >81.0%, Group 4: >85.0%, Group 5: >86.0%, Group 6: >90.0%, and Group 7: >70.0%) than those of the feature vectors with low inter-session PCCs (*r* < 0.7). The feature vectors, which were used in the feature combination from previous studies, exhibited high inter-session PCCs (*r* > 0.7).

### 3.3. Classification Accuracy and Inter-Feature PCC

**Figure 5:**
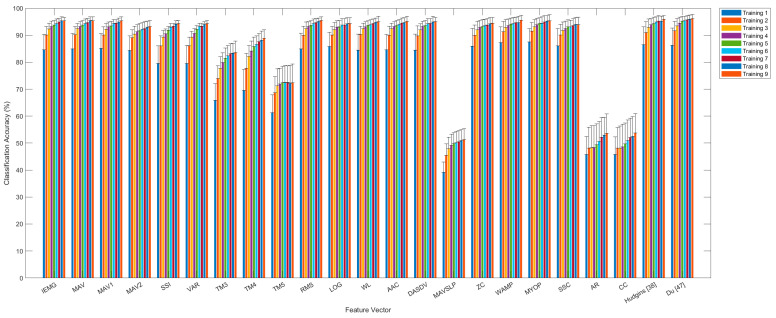
Classification accuracies of feature vectors and feature combinations according to training in Group 1.

**Figure 6:**
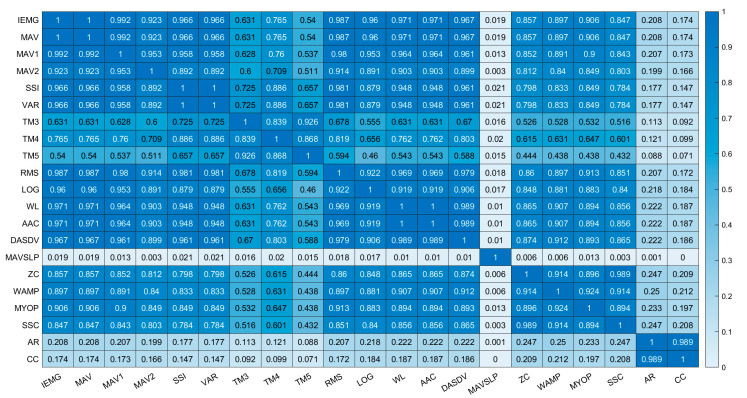
Confusion matrix of inter-feature PCC in Group 1.

The classification accuracies of all of the feature vectors and their combinations in Group 1 are presented in Figure 5 (the results for the other groups are presented in Figures S13–S18). The classification accuracies of all feature vectors, including the combinations of feature vectors, were improved by increasing the number of training sessions. However, certain feature vectors, such as MAVSLP, AR and CC, demonstrated lower classification accuracy (51.3%~53.7%) and inter-session PCC (*r* < 0.3) than other feature vectors, even though nine training sessions were applied. On the contrary, the feature vectors with a high inter-session PCC (*r* > 0.7) showed a classification accuracy of higher than 90.0%, which was statistically similar with the classification accuracy of the feature vector combinations. Furthermore, Figure 6 and Figures S19–S24 showed that strong linear relationship in the inter-feature PCC appeared between the feature vectors with a high inter-session PCC (*r* > 0.7), while a weak linear relationship in the inter-feature PCC appeared between the feature vectors with a high inter-session PCC (*r* > 0.7) and the feature vectors with a low inter-session PCC (*r* < 0.3). These results reveal that information was similar between the feature vectors that had a high inter-session PCC and high classification accuracy.

### 3.4. Classification Accuracies According to Hand Posture Groups

**Figure 7:**
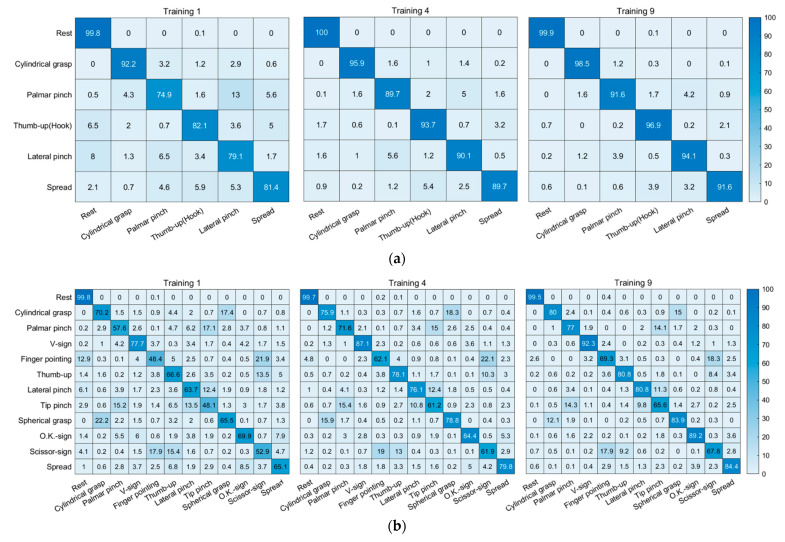
Confusion matrices of the hand postures: (**a**) Group 1 and (**b**) Group 7.

Figure 7 shows the confusion matrices of the Group 1 and Group 7 in MAV, which is a well-known feature vector (all results are presented in Figures S25–S31). In Group 1, the hand gestures of rest and cylindrical grasp showed the high classification accuracy, while the worst classification accuracy appeared in palmar pinch. The misclassification between palmar pinch and lateral pinch was also minimized from 13.0% to 4.2% by increasing the number of training sessions. The change in the misclassification in relation to the number of training sessions in Group 2 were from 12.6% to 4.0% and 14.4% to 3.7% for ‘palmar pinch vs. lateral pinch’ and ‘finger pointing vs. rest’, respectively (Figure S26). However, the misclassifications of ‘palmar pinch vs. tip pinch vs. lateral pinch’, ‘cylindrical grasp vs. spherical grasp’, and ‘scissor sign vs. thumb up (hook)’ were still high despite the increasing number of training sessions in Groups 3–5 (Figures S27–S29). Group 6 (Figure S30) exhibited a good classification performance independent of the number of training sessions. Group 7 revealed all types of misclassifications. As shown in the confusion matrices, the hand postures of a similar form or function showed more misclassifications, as they were more difficult to differentiate from one other. However, most misclassifications were significantly reduced given an increasing number of training sessions. The same results were obtained in the other feature vectors.

## 4. Discussion

This study was conducted to develop an sEMG-based hand posture recognition system that takes into consideration three problems: electrode shift, feature vector selection, and hand posture selection. The classification accuracy, which is negatively affected by the electrode shift and misclassification between similar postures, was improved by increasing the number of training sessions and selecting hand postures with a consideration of their functions and the associated activated muscles. Furthermore, an efficient feature vector optimization method was developed by analyzing the relationship between the classification and inter-session PCC. These findings provide a method for developing an sEMG-based hand posture recognition system displaying a high practicality.

Electrode positional changes are common in sEMG-based gesture recognition systems in daily life; however, many previous studies reported that the positions of the sEMG sensors were fixed to avoid the occurrence of misclassification resultant of the electrode shift. Lu et al. used seven sEMG sensors with fixed positions on the muscle belly to control a robotic hand [48]. The measured sEMG signal was applied to a Gaussian Naive Bayes classifier and an SVM, and six hand gestures were classified with an accuracy of 84.1%. sEMG-based gesture recognition algorithms with fixed sEMG sensor positions are appropriate for robotic prosthesis, which uses a socket for preventing the electrode shift; however, it is not appropriate in the HCI interface for non-expert users. Phinyomark et al. emphasized the importance of the solution for the confounding factors, such as the electrode shift, to improve its re-usability and sustainability for real-world application with long-term use [49]. These confounding factors were addressed by a big dataset that was measured over a long-term experiment using the electrode shift. 

In this study, an armband sensor was designed to measure the EMG signal pattern, which is related to the hand gesture of the user, even if the individual sensor is not placed on the specific muscles. Furthermore, the position of the main module was only suggested for non-expert users, and the experiment was repeated 10 times with the donning and doffing of the armband sensor to induce the electrode shift. Relatively low classification accuracies appeared in all posture groups with one training session (in MAV, Group 1: 84.9 ± 5.8%, Group 2: 80.9 ± 6.6%, Group 3: 75.5 ± 7.9%, Group 4: 79.2 ± 7.8%, Group 5: 79.5 ± 6.4% Group 6: 83.6 ± 8.6%, and Group 7: 65.5 ± 10.2%) owing to a lack of training on the electrode shift. The highest classification accuracies appeared after nine training sessions (in MAV, Group 1: 95.4 ± 1.4%, Group 2: 93.6 ± 2.2%, Group 3: 87.6 ± 3.6%, Group 4: 91.4 ± 3.6%, Group 5: 91.3 ± 3.7%, Group 6: 95.5 ± 2.0%, and Group 7: 80.9 ± 5.2%, respectively). Hence, in all feature vectors and posture groups, the classification accuracy improved with an increasing number of training sessions for the electrode shift, and a statistically significant improvement was observed when at least three training sessions were conducted. The classification accuracy improved when more than four training sessions were conducted, but the difference was not as significant. These results indicate that the problem of classification accuracy degradation could be resolved by increasing the number of training sessions on the electrode shift; the number of training sessions for efficiency was four.

Oskoei et al. reported that feeding a myoelectric signal presented as a time sequence directly to a classifier is impractical because of the large number of inputs and the randomness of the signal [50]. Therefore, many previous studies used feature vectors to compress the data and to normalize the pattern. Most of the previous studies selected the feature vectors according to the results of the classification performance evaluation [9,51,52]; however, accuracy-based feature vectors selection requires significant time and high costs because the pattern recognition algorithms are affected by various confounding factors. Data processing techniques, such as PCA and GA, were suggested to improve the classification performance of the feature vectors. Kakoty et al. reported that the classification accuracy improved by more than 8.0% through the compressed information from the PCA-based dimension reduction [12], which was useful for reducing the complexity of data or to reinforce the important information for pattern recognition, but the process of selecting the feature vector still remains unsolved. Oskoei et al. proposed a method for selecting feature vectors using entropy and GA [32,50], and Phinyomark et al. reported an RES-index-based feature vector selection, based on Euclidean distance and standard deviation [12]. These methods of selecting feature vectors are efficient because the classification performance of each feature vector can be evaluated before the development of the classifiers; however, few studies have been conducted on feature vector selection considering the electrode shift.

Twenty-one feature vectors were selected in the time domain, considering the real-time application in this study. These feature vectors were applied to the ANN-based classifier as a single type, and the classification performance of each feature vector was analyzed with the inter-session and inter-feature PCCs. The results indicate that the feature vectors with a strong linear relationship in inter-session PCC (*r* > 0.7) had a higher classification accuracy than that of the feature vectors with low inter-session PCC (*r* < 0.7), and these results were obtained for all training conditions and posture groups. Furthermore, in the threshold-based feature vectors of ZC, WAMP, MYOP, and SSC, the appropriate ranges of threshold values were found from the high inter-session PCC (*r* > 0.8). These results indicate that the inter-session PCC was well-correlated with the classification accuracy of each feature vector, and the feature vectors and the threshold values could be selected easily and efficiently by analyzing the inter-session PCC.

There were two differences in the feature vectors between this study and previous studies. The first difference was in the classification accuracies of AR and CC, and the second was in the effects of the feature combinations. AR and CC, which are calculated by the auto-regressive model, are well known as feature vectors with excellent classification performances [53]. However, AR and CC exhibited the lowest classification accuracies in this study, owing to the lack of data to calculate the feature vectors. In this study, the amount of data available for calculating the feature vectors was less than for previous studies because the armband sensor had a sampling rate of 500 Hz, whereas previous studies used sEMG sensors with a sampling rate of at least 1000 Hz. Phinyomark et al. evaluated the gesture recognition algorithm using various sEMG datasets and reported that the classification performances of AR and CC degraded owing to a lack of data points [54]. The problem resulting from the lack of data could be solved by increasing the sampling rate, but this solution will lead to an increase in the computational load and power consumption in the HCI interface and wearable device that is used over a long period in daily life. Increasing the window size used to calculate the feature vectors is another solution for the lack of data, but this solution is not thought to be appropriate because the increased window size introduces a delay in the feature vector calculation process. Therefore, AR and CC are inappropriate in the HCI interface and wearable device, as they set high values for efficiency and real-time classification.

Feature combinations were applied to improve the classification performance of pattern recognition algorithms in previous studies, with Hudgins’ set [38] and Du’s set [47] being the main combinations used. Hudgins’ set comprises MAV, WL, ZC, and SSC whereas Du’s set comprises IEMG, VAR, WL, ZC, SSC, and WAMP. Among the feature vectors in these combinations, those of ZC, WAMP, and SSC are well known to possess the frequency information, although these are included in the time domain [53] and are useful for gathering various information in real-time applications. Phinyomark et al. compared the classification performances of the single feature vectors and feature combinations (Hudgins’ set and Du’s set) and reported that the latter is superior [53]. However, they also reported that the difference in classification accuracy between individual feature vectors with high inter-session PCCs (*r* > 0.7) and the feature combinations is not significant. These results were obtained using the sampling rate of the sensor and noise from the crosstalk via the electrode shift. Many previous studies that reported improved classification performances with feature combinations used sEMG sensors with a high sampling rate (approximately 1000 Hz) and fixed positions on specific muscles. These experimental methods had the advantages of avoiding noise from crosstalk and gathering enough frequency information with the calculation of ZC, WAMP, and SSC; however, in this study, the frequency information was not sufficient in the ZC, WAMP, and SSC, owing to the low sampling frequency (500 Hz) and noise from the crosstalk. Furthermore, the inter-feature PCC between feature vectors with a high inter-session PCC (*r* > 0.7) was higher than 0.8 (strong linear relationship). These results indicate that these feature vectors (even the ZC, WAMP, and SSC) had the same information. Therefore, the classification performance of the feature combinations degraded. These problems, such as the degradation of the classification performance by the crosstalk, could be solved by fixing the position of the electrode, but finding the position for each specific muscle is difficult for non-expert users in daily life. Additional sensors, such as the IMU, will help provide diverse information on the feature combinations to improve the classification performance.

For the development of a gesture recognition algorithm, many studies have applied various groups of gestures and postures based on their research aim. However, the classification accuracy degraded with similar gestures in a gesture group because the function alone of each gesture was considered. Andrade et al. selected six gestures—cylindrical grasp, tip pinch, hook (snap), palmar pinch, spherical grasp, and lateral pinch—to develop an sEMG-based gesture recognition algorithm [21]. Their results indicated that the misclassification for similar gestures that are activated by the same muscle (precision grasp: tip pinch, palmar pinch, and lateral pinch; power group: cylindrical grasp, hook, and spherical grasp) was high for the gesture recognition algorithm.

In this study, twelve hand postures—which are frequently used and are important in hand posture recognition algorithms—were selected after a literature review. Seven different posture groups were formed according to the functions and activated muscles of hand postures to analyze the effects of similar postures. In Andrade’s study, the rate of misclassification between similar postures was high, and this misclassification was not addressed in various feature vectors with an increase in the number of training sessions. The classification accuracies were in the following order: Group 1 (six hand postures) > Group 6 (seven hand postures considering the function and the muscle activation) > Group 2 (Group 1 + finger pointing) > Group 4 (Group 1 + similar posture: spherical grasp) ≥ Group 5 (Group 1 + similar posture: scissor-sign) > Group 3 (Group 1 + similar posture: tip pinch) > Group 7 (twelve hand postures). For Groups 2 to 6, the number of postures was 7, but the classification accuracies differed with the addition of postures (similar or not). More specifically, Group 3 exhibited the lowest classification accuracy because of the tip pinch posture, which was similar to both the palmar pinch and lateral pinch. Groups 2 and 6 were similar in that the postures were selected with consideration to the activated muscles. Group 2 had the spread posture and Group 6 had the V-sign posture. Group 6 had a higher classification accuracy than Group 2, but the difference was not significant. Furthermore, the spread posture, which opens the hand and supports loads, is more useful than the V-sign posture, which expresses emotions. Hence, Group 2 is more efficient in the HCI interface. These results indicate that the development of the gesture recognition algorithm could be made more efficient by the selection of gestures, giving consideration to both functions and the activated muscles.

This study has three limitations. The first is related to pattern information. All EMG feature vectors were selected in the time domain considering real-time application. In addition, because the armband sensor had a lower sampling frequency than that of the sEMG sensor in the previous study, the feature vectors information had to be similar. The second limitation is that the hand posture recognition algorithm was optimized for each subject, but normalization did not occur for optimization in all subjects. The gesture recognition algorithm without the normalization required more time and a higher cost because the classifier had to be trained for each user. The last limitation is that the gestures of this study were static postures only, and dynamic gestures were not considered.

## 5. Conclusions

This paper presented an sEMG-based hand posture recognition algorithm using an armband sensor, considering the following three problems: electrode shift, feature vector selection, and postures selection. This study showed that the electrode shift could degrade the classification performance of the pattern recognition algorithm, and this problem could be solved by increasing the number of training sessions on the electrode shift. Additionally, the inter-session PCC was verified as a means for selecting feature vectors because it exhibits a strong relationship with feature vectors and threshold values, with a high classification accuracy. Furthermore, information on each feature vector was compared with the inter-feature PCC, and the results of this analysis confirm that an additional sensor, such as an IMU, is required to provide diverse information for improving the hand posture recognition algorithm. In addition, selecting the target postures with consideration to the functions and activated muscles was as important as selecting feature vectors with a high classification accuracy for the development of an efficient posture recognition algorithm. These findings will be helpful in assisting the development process of sEMG-based gesture recognition algorithms more efficiently. In future works, the IMU sensor and the normalized algorithm will be applied in the pattern recognition system to provide diverse information and to reduce the training time and associated costs. Furthermore, the reaction speed and the practicality of the pattern recognition algorithm will be improved via the recognition of dynamic gestures to expand the application range.

## Figures and Tables

**Table 1:** Hand posture functions and groups.

Function	Hand Postures			Posture Groups	
Holding objects	Cylindrical grasp [21,27,28] (for cylindrical objects)	Spherical grasp [21,28](for spherical objects)		Group 1	Rest, cylindrical grasp, palmar pinch, hook (thumb-up), lateral pinch, spread
				
Holding small/thin/flat objects	Palmar pinch [21,28] (using thumb, index, and middle fingers for palm facing the objects)	Tip pinch [21,28] (using thumb and index fingers for palm facing the objects)		Group 2	Group 1 + finger pointing
	Lateral pinch [21,27] (using a thumb pad and the radial side of the index finger)			Group 3	Group 1 + tip pinch
				Group 4	Group 1 + spherical grasp
Releasing objects	-	Spread [10,24,27,29,30]		Group 5	Group 1 + scissor-sign
Supporting loads					
Expression of emotion	Thumb-up (hook) [10,21,23,28,29,30]	V-sign [10,25,29,30]	O.K.-sign [10,22,29,30]	Group 6	Rest, cylindrical grasp, palmar pinch, V-sign, finger pointing, thumb-up (hook), lateral pinch
No activation	Rest				
Pointing objects	Finger pointing [10,23,27] (using index finger, only)	Scissor-sign [23,26] (using thumb and index finger)			
				Group 7	All hand postures (12)

Hand postures with gray background: the most frequently used and important hand postures in previous studies.

**Table 2:** Time-domain feature vectors.

*N*: window size, *i*: data sample, *EMG_i_*: sEMG signal, *w_i_*: white noise error term; *p*: function order			
$RMS=\sqrt{\frac{1}{N}\overset{N}{\underset{i=1}{\sum }}EMG_{i}{}^{2}}$	$WL=\overset{N-1}{\underset{i=1}{\sum }}\left|EMG_{i+1}-EMG_{i}\right|$	$MAV=\frac{1}{N}\overset{N}{\underset{i=1}{\sum }}\left|EMG_{i}\right|$	$MAVSLP_{i}=MAV_{i+1}-MAV_{i}$
$MAV1\;\&\;MAV2=\frac{1}{N}\overset{N}{\underset{i=1}{\sum }}w_{i}\left|EMG_{i}\right|\;MAV1:\;w_{i}=\{\begin{array}{c} 1,\;if\;0.25N\leq i\leq 0.75N\\ 0.5,\;\;\;\;\;\;\;\;\;\;\;\;\;\;\;\;\;\;\;otherwise \end{array}\;MAV2:\;w_{i}=\{\begin{array}{c} 1,\;if\;0.25N\leq i\leq 0.75N\\ \frac{4i}{N},\;\;elseif\;i<0.25N\\ \frac{4\left(i-N\right)}{N},\;\;otherwise \end{array}$		$ZC=\overset{N-1}{\underset{i=1}{\sum }}\left[f\left(x_{i}\times x_{i+1}\right)\cap \left|x_{i}-x_{i+1}\right|\geq threshold\right]$	
		$WAMP=\overset{N-1}{\underset{i=1}{\sum }}\left[f\left(\left|x_{i}-x_{i+1}\right|\right)\right]$	$f\left(x\right)=\{\begin{array}{c} 1,\;if\;x\geq threshold\\ 0,\;\;\;\;\;\;\;\;\;\;\;\;\;\;otherwise \end{array}$
		$SSC=\overset{N-1}{\underset{i=2}{\sum }}\left[f\left[\left(x_{i}-x_{i-1}\right)\times \left(x_{i}-x_{i+1}\right)\right]\right]$	
$IEMG=\overset{N}{\underset{i=1}{\sum }}\left|EMG_{i}\right|$	$VAR=\frac{1}{N-1}\overset{N}{\underset{i=1}{\sum }}EMG_{i}{}^{2}$	$SSI=\overset{N}{\underset{i=1}{\sum }}EMG_{i}{}^{2}$	$DASDV=\sqrt{\frac{1}{N-1}\overset{N-1}{\underset{i=1}{\sum }}{\left(x_{i+1}-x_{i}\right)}^{2}}$
$TM3=\left|\frac{1}{N}\overset{N}{\underset{i=1}{\sum }}EMG_{i}{}^{3}\right|\;TM4=\frac{1}{N}\overset{N}{\underset{i=1}{\sum }}EMG_{i}{}^{4}\;TM5=\left|\frac{1}{N}\overset{N}{\underset{i=1}{\sum }}EMG_{i}{}^{5}\right|$	$LOG=e^{\frac{1}{N}\sum_{i=1}^{N}log\left(\left|EMG_{i}\right|\right)}$	$MYOP=\frac{1}{N}\overset{N}{\underset{i=1}{\sum }}\left[f\left(x_{i}\right)\right]$	$AAC=\frac{1}{N}\overset{N-1}{\underset{i=1}{\sum }}\left|x_{i+1}-x_{i}\right|$
	$AR=a_{p}$; $Auto-regressive\;mode:\;x_{i}=\sum_{p=1}^{P}a_{p}x_{i-p}+w_{i}$		
	$CC=c_{p}$; $c_{1}=-a_{1}$; $c_{p}=-a_{p}-\sum_{l=1}^{p-1}\left(1-\frac{l}{p}\right)a_{p}c_{p-l}$; 1≤l≤p		

**Table 3:** Classification accuracy and inter-session PCC of the feature vectors in Group 1.

Feature Vector	Classification Accuracy (%): Mean (Standard Deviation)																		PCC (*r*)
	TRN1		TRN2		TRN3		TRN4		TRN5		TRN6		TRN7		TRN8		TRN9		
IEMG	84.6	(5.7)	90.1	(3.1)	92.3	(2.2)	93.2	(2.0)	93.8	(1.7)	94.4	(1.6)	94.9	(1.3)	95.4	(1.4)	95.4	(1.2)	0.837
MAV	84.9	(5.8)	90.2	(3.0)	92.5	(1.9)	93.2	(1.8)	93.8	(1.7)	94.3	(1.7)	94.7	(1.5)	95.5	(1.3)	95.4	(1.4)	0.837
MAV1	85.2	(5.5)	90.1	(3.0)	92.2	(2.1)	93.1	(1.8)	93.5	(1.6)	94.3	(1.5)	94.5	(1.3)	94.9	(1.5)	95.4	(1.4)	0.835
MAV2	84.4	(5.3)	89.1	(2.9)	90.4	(2.9)	91.3	(2.6)	91.7	(2.7)	92.2	(2.5)	92.5	(2.5)	93.0	(2.3)	93.2	(2.3)	0.808
SSI	79.6	(6.5)	86.0	(3.8)	89.3	(2.5)	90.5	(1.8)	91.9	(1.3)	93.0	(1.3)	93.3	(1.1)	94.1	(1.3)	94.4	(1.1)	0.735
VAR	79.5	(6.7)	86.0	(3.3)	89.3	(2.1)	90.6	(1.8)	92.2	(1.4)	93.2	(1.3)	93.3	(1.2)	94.0	(1.0)	94.3	(1.2)	0.735
TM3	65.9	(6.1)	73.9	(4.7)	77.7	(4.3)	79.9	(3.6)	81.5	(3.8)	82.4	(3.8)	83.1	(3.7)	83.4	(3.5)	83.6	(4.2)	0.392
TM4	69.6	(7.7)	77.8	(5.5)	82.1	(4.1)	84.1	(3.7)	85.8	(3.5)	86.6	(3.1)	87.6	(3.1)	88.2	(3.4)	88.9	(3.1)	0.428
TM5	61.3	(6.6)	68.7	(5.9)	71.3	(6.2)	71.9	(5.8)	72.4	(6.0)	72.5	(6.3)	72.5	(6.1)	72.1	(6.7)	72.4	(6.9)	0.248
RMS	84.9	(5.9)	90.0	(3.3)	92.4	(2.5)	93.2	(1.8)	93.7	(1.7)	94.3	(1.7)	94.7	(1.4)	95.1	(1.3)	95.5	(1.3)	0.829
LOG	85.8	(5.3)	90.1	(3.1)	92.1	(2.6)	93.0	(2.4)	93.1	(2.4)	93.8	(2.3)	93.8	(2.4)	94.2	(2.3)	94.3	(2.2)	0.859
WL	84.5	(5.9)	90.3	(3.0)	92.4	(2.1)	93.1	(1.9)	93.7	(1.7)	93.9	(1.8)	94.4	(1.6)	94.8	(1.6)	95.0	(2.1)	0.832
AAC	84.6	(5.7)	90.0	(3.3)	92.4	(2.1)	93.2	(1.8)	93.6	(1.9)	94.0	(1.8)	94.5	(1.9)	94.7	(1.8)	95.1	(2.0)	0.832
DASDV	84.5	(5.9)	89.8	(3.7)	92.0	(2.2)	93.2	(1.9)	93.6	(1.9)	94.2	(1.9)	94.3	(1.8)	94.9	(2.1)	95.0	(1.6)	0.824
MAVSLP	39.2	(3.8)	45.5	(4.2)	47.9	(4.3)	49.1	(4.2)	49.8	(4.1)	50.2	(4.2)	50.5	(4.2)	50.9	(4.1)	51.3	(4.0)	0.005
ZC	85.9	(6.7)	90.0	(3.8)	92.0	(3.1)	92.9	(2.6)	93.2	(2.6)	93.6	(2.3)	93.8	(2.3)	94.2	(2.4)	94.5	(2.1)	0.858
WAMP	87.3	(5.8)	91.3	(3.8)	92.9	(2.9)	93.6	(2.4)	94.1	(2.3)	94.3	(2.3)	94.7	(2.0)	94.7	(2.3)	95.6	(1.8)	0.873
MYOP	87.5	(5.0)	91.5	(3.2)	93.1	(2.7)	93.9	(2.4)	94.4	(2.1)	94.5	(2.3)	94.9	(2.4)	95.2	(2.1)	95.4	(2.2)	0.876
SSC	86.0	(6.5)	90.1	(3.9)	91.8	(3.1)	92.4	(2.8)	93.1	(2.6)	93.1	(2.5)	93.6	(2.5)	94.0	(2.6)	93.9	(2.8)	0.862
AR	45.8	(6.6)	48.1	(7.8)	48.3	(8.2)	48.4	(8.0)	49.4	(7.7)	50.6	(7.4)	52.2	(7.3)	53.0	(6.6)	53.6	(7.2)	0.278
CC	45.8	(6.5)	48.2	(7.6)	48.1	(8.2)	48.6	(8.4)	49.7	(7.8)	50.9	(7.7)	52.1	(7.1)	52.5	(7.3)	53.7	(7.2)	0.299

## Data Availability

The data presented in this study are available on request from the corresponding author. The data are not publicly available owing to continuing study by the authors.

## Supplementary Materials

The following are available online at https://www.mdpi.com/article/10.3390/s21227681/s1, Figure S1: Classification accuracy and inter-session PCC of Group 2 by threshold values; Figure S2. Classification accuracy and inter-session PCC of Group 3 by threshold values; Figure S3. Classification accuracy and inter-session PCC of Group 4 by threshold values; Figure S4. Classification accuracy and inter-session PCC of Group 5 by threshold values; Figure S5. Classification accuracy and inter-session PCC of Group 6 by threshold values; Figure S6. Classification accuracy and inter-session PCC of Group 7 by threshold values; Figure S7. Classification accuracy and inter-session PCC of Group 2 by feature vector order; Figure S8. Classification accuracy and inter-session PCC of Group 3 by feature vector order; Figure S9. Classification accuracy and inter-session PCC of Group 4 by feature vector order; Figure S10. Classification accuracy and inter-session PCC of Group 5 by feature vector order; Figure S11. Classification accuracy and inter-session PCC of Group 6 by feature vector order; Figure S12. Classification accuracy and inter-session PCC of Group 7 by feature vector order; Figure S13. Classification accuracy of feature vectors and combinations by number of training sessions in Group 2; Figure S14. Classification accuracy of feature vectors and combinations by number of training sessions in Group 3; Figure S15. Classification accuracy of feature vectors and combinations by number of training sessions in Group 4; Figure S16. Classification accuracy of feature vectors and combinations by number of training sessions in Group 5; Figure S17. Classification accuracy of feature vectors and combinations by number of training sessions in Group 6; Figure S18. Classification accuracy of feature vectors and combinations by number of training sessions in Group 7; Figure S19. Confusion matrix of inter-feature PCC in Group 2; Figure S20. Confusion matrix of inter-feature PCC in Group 3; Figure S21. Confusion matrix of inter-feature PCC in Group 4; Figure S22. Confusion matrix of inter-feature PCC in Group 5; Figure S23. Confusion matrix of inter-feature PCC in Group 6; Figure S24. Confusion matrix of inter-feature PCC in Group 7; Figure S25. Confusion matrix of the hand postures of Group 1; Figure S26. Confusion matrix of the hand postures of Group 2; Figure S27. Confusion matrix of the hand postures of Group 3; Figure S28. Confusion matrix of the hand postures of Group 4; Figure S29. Confusion matrix of the hand postures of Group 5; Figure S30. Confusion matrix of the hand postures of Group 6; Figure S31. Confusion matrix of the hand postures of Group 7; Table S1. Classification accuracy and inter-session PCC of the feature vectors in Group 2; Table S2. Classification accuracy and inter-session PCC of the feature vectors in Group 3; Table S3. Classification accuracy and inter-session PCC of the feature vectors in Group 4; Table S4. Classification accuracy and inter-session PCC of the feature vectors in Group 5; Table S5. Classification accuracy and inter-session PCC of the feature vectors in Group 6; Table S6. Classification accuracy and inter-session PCC of the feature vectors in Group 7.
