# Supplementary material for: sEMG-Based Hand Posture Recognition Considering Electrode Shift, Feature Vectors, and Posture Groups
Source: Sensors (Basel). 2021 Nov 18;21(22):7681. doi: 10.3390/s21227681 (PMC8624257; doi:10.3390/s21227681)
Supplement: Supplementary file 1 [file sensors-21-07681-s001.zip › sensors-1447945-supplementary.pdf]

sensors

Article

sEMG-Based Hand Posture Recognition Considering Electrode Shift, Feature Vectors, and Posture Groups

Jongman Kim, Bummo Koo, Yejin Nam and Youngho Kim *

Department of Biomedical Engineering, Yonsei University, Wonju 26493, Korea; jmkim0127@ybrl.yonsei.ac.kr (J.K.); bmk726@ybrl.yonsei.ac.kr (B.K.); namyj_1007@naver.com (Y.N.)

* Correspondence: younghokim@yonsei.ac.kr; Tel.: +82-33-760-2859

Supplementary Materials:

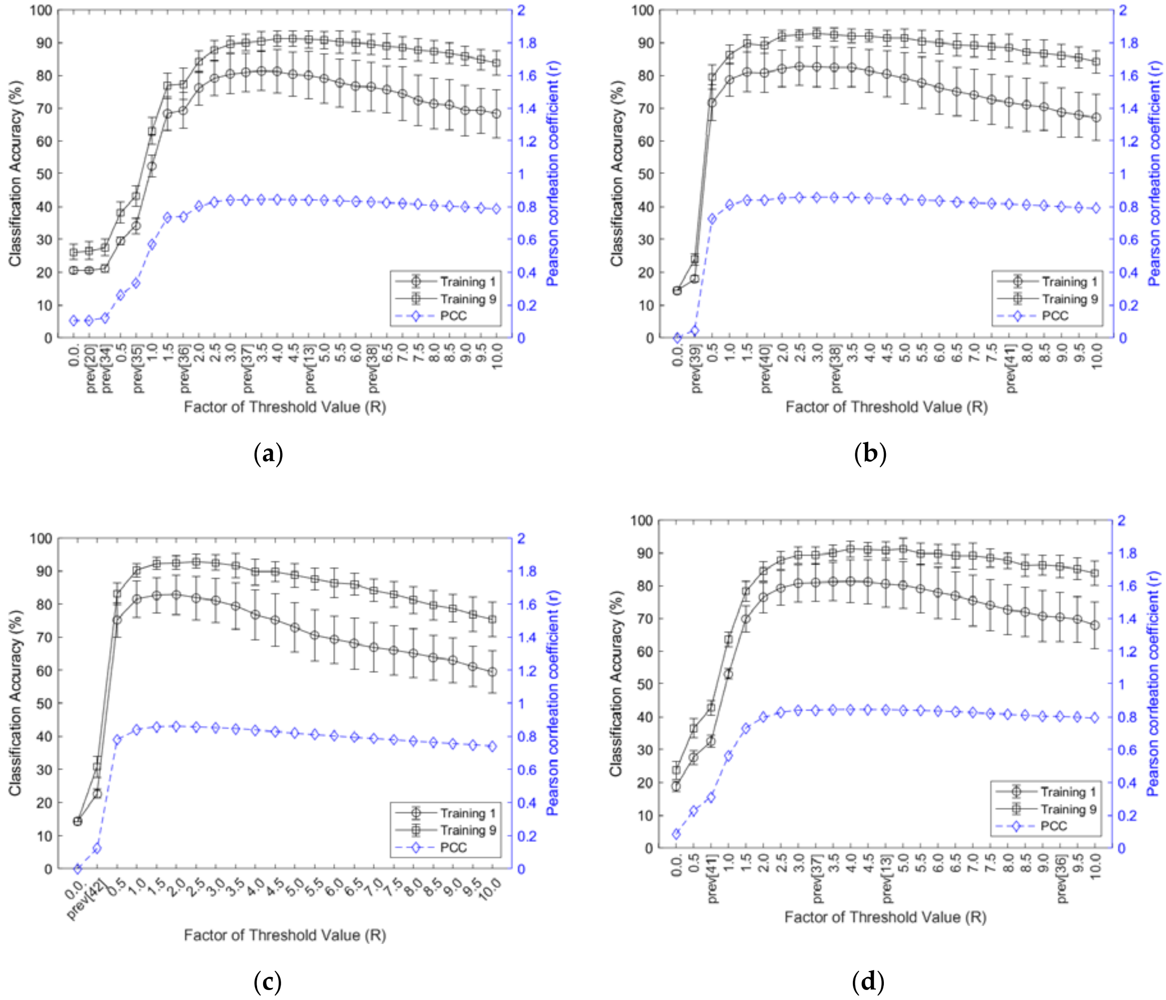

Figure S1. Classification accuracy and inter-session PCC of Group 2 by threshold values: (**a**) ZC; (**b**) WAMP; (**c**) MYOP; (**d**) SSC; prev: threshold value from the previous study.

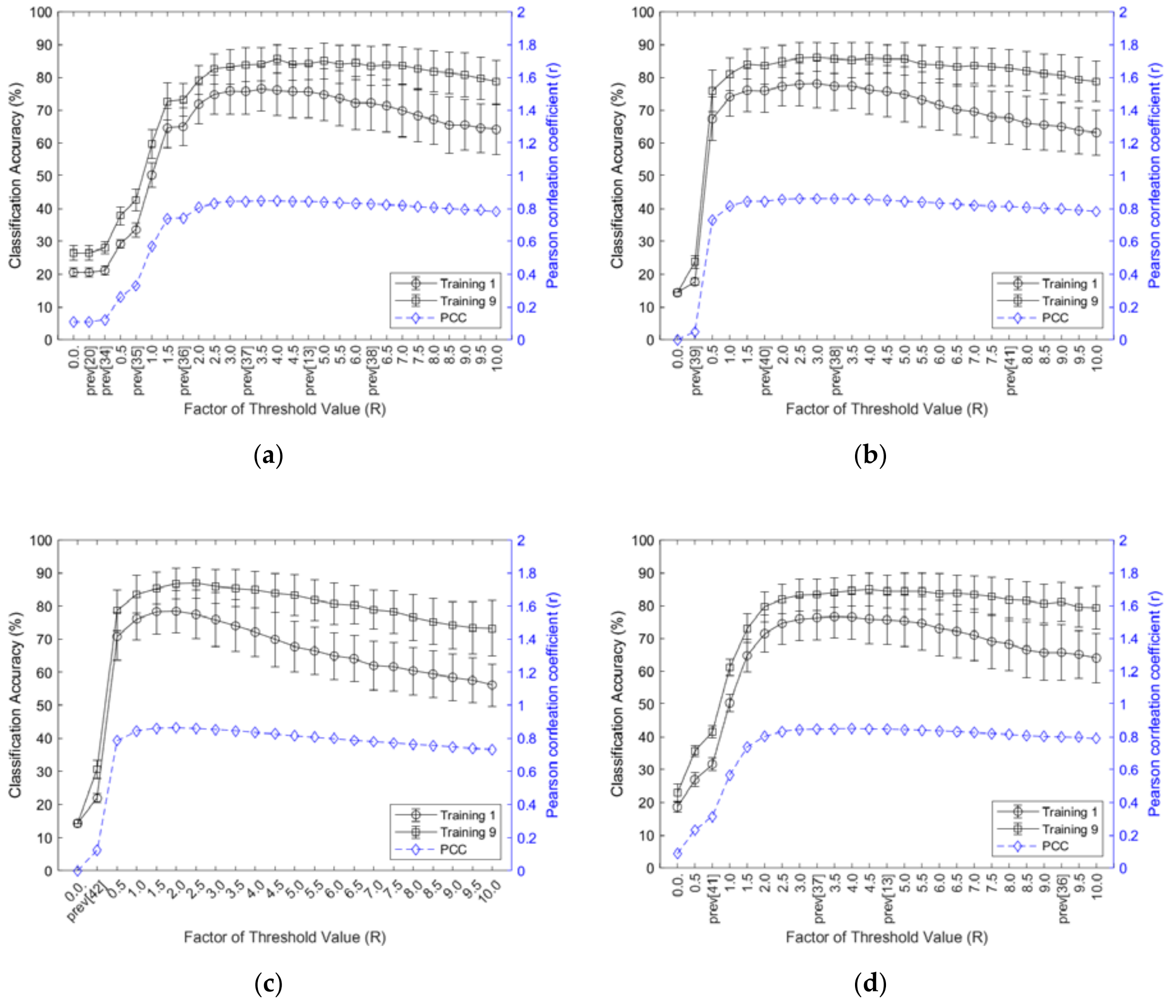

Figure S2. Classification accuracy and inter-session PCC of Group 3 by threshold values: (**a**) ZC; (**b**) WAMP; (**c**) MYOP; (**d**) SSC; prev: threshold value from the previous study.

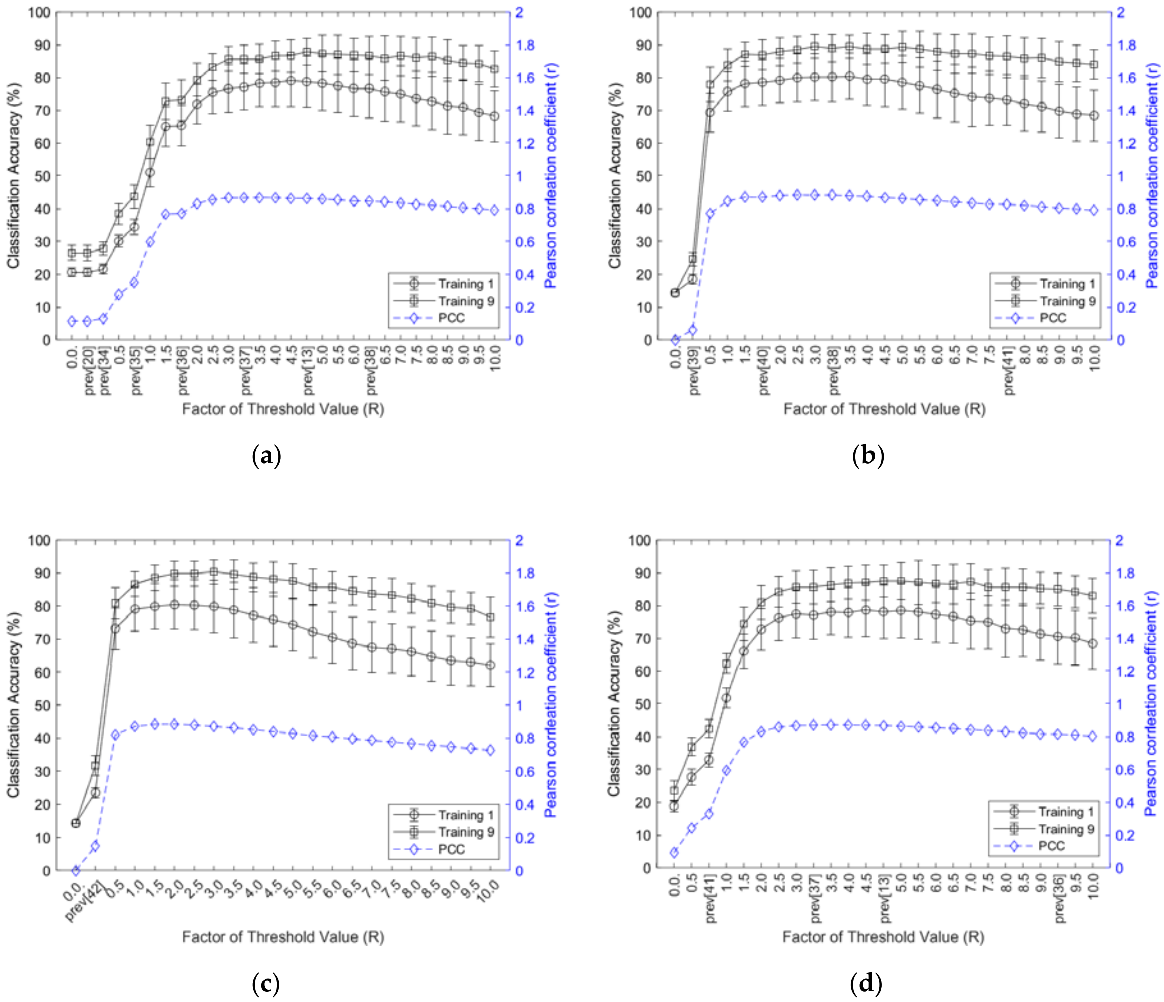

Figure S3. Classification accuracy and inter-session PCC of Group 4 by threshold values: (**a**) ZC; (**b**) WAMP; (**c**) MYOP; (**d**) SSC; prev: threshold value from the previous study.

sensors

MDPI

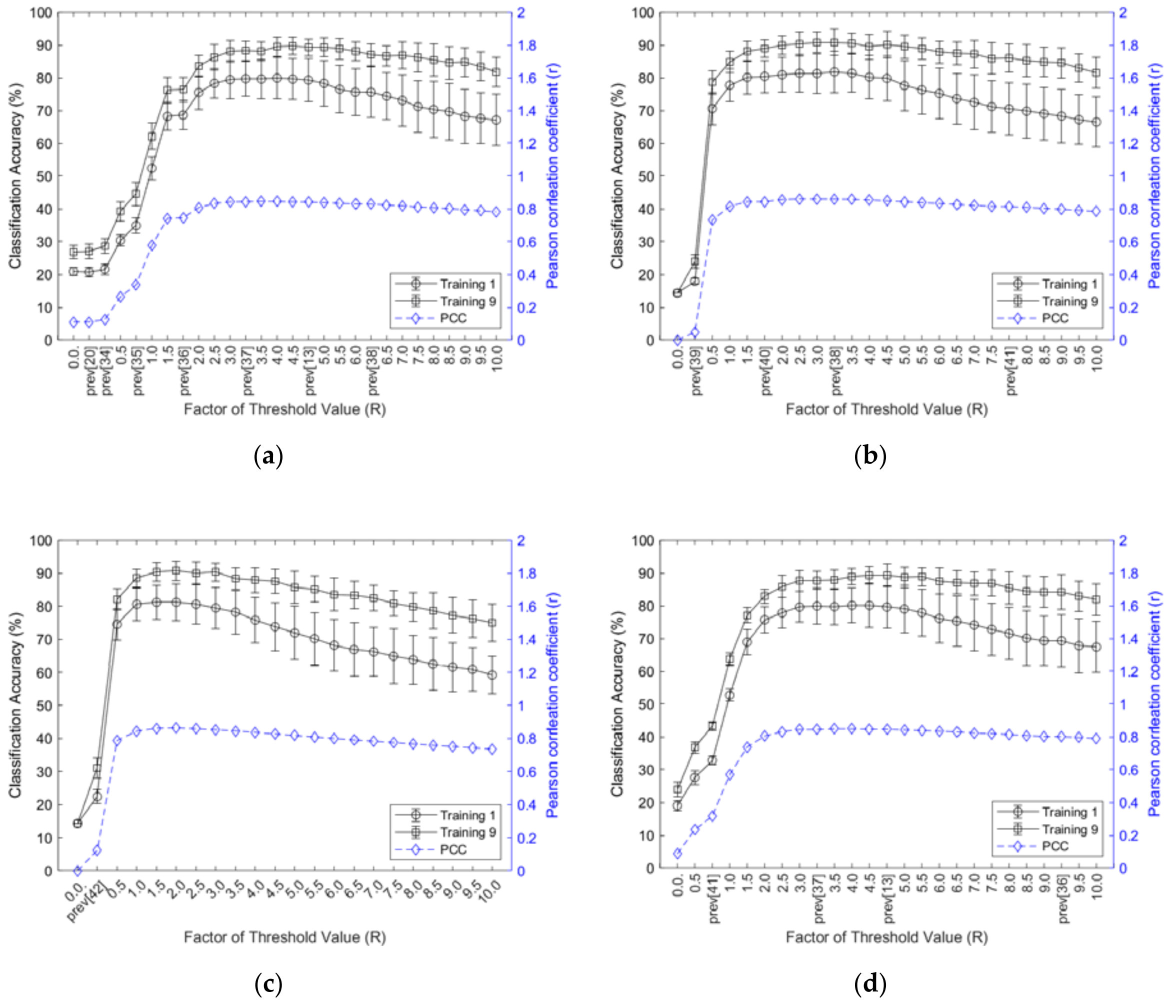

Figure S4. Classification accuracy and inter-session PCC of Group 5 by threshold values: (**a**) ZC; (**b**) WAMP; (**c**) MYOP; (**d**) SSC; prev: threshold value from the previous study

sensors

MDPI

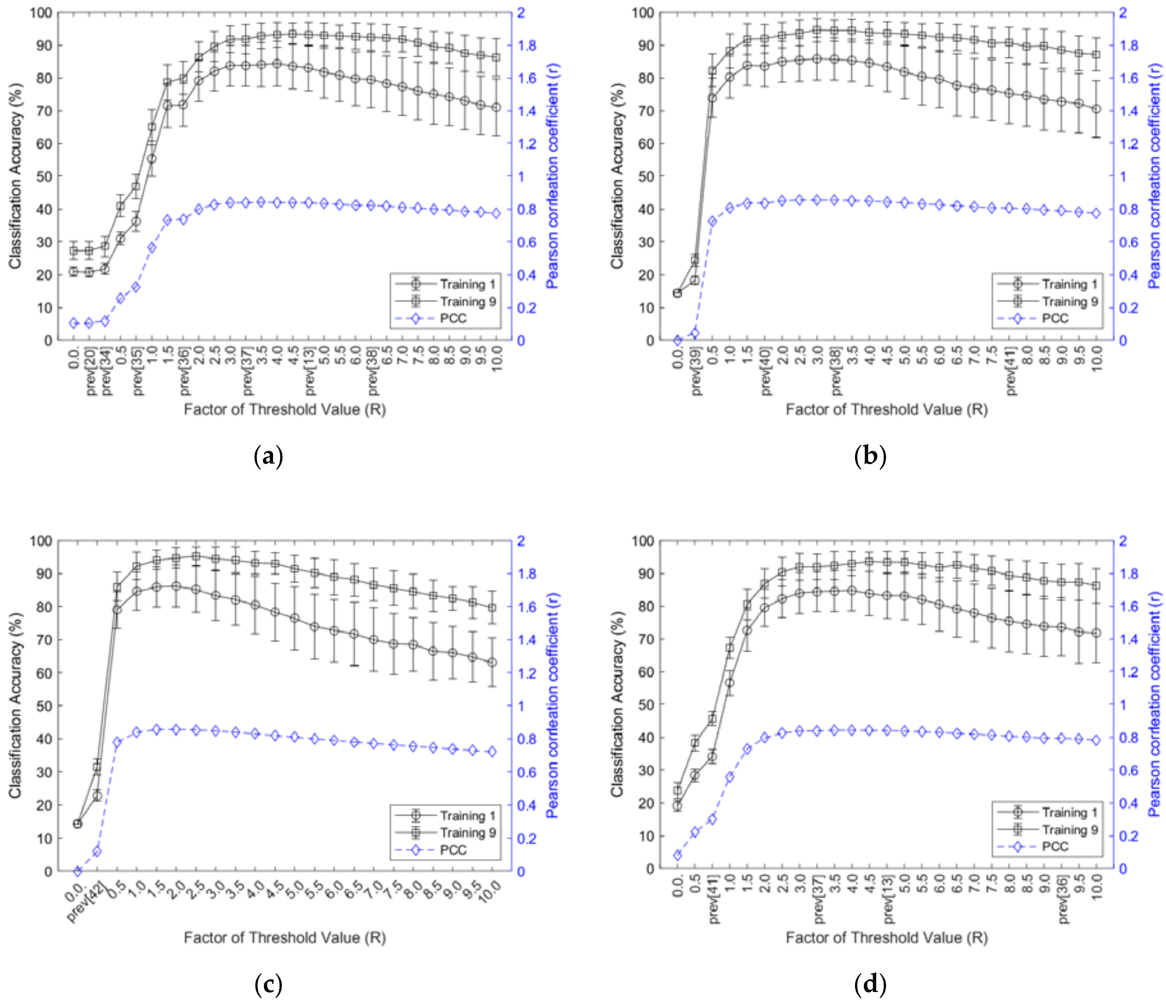

Figure S5. Classification accuracy and inter-session PCC of Group 6 by threshold values: (a) ZC; (b) WAMP; (c) MYOP; (d) SSC; prev: threshold value from the previous study.

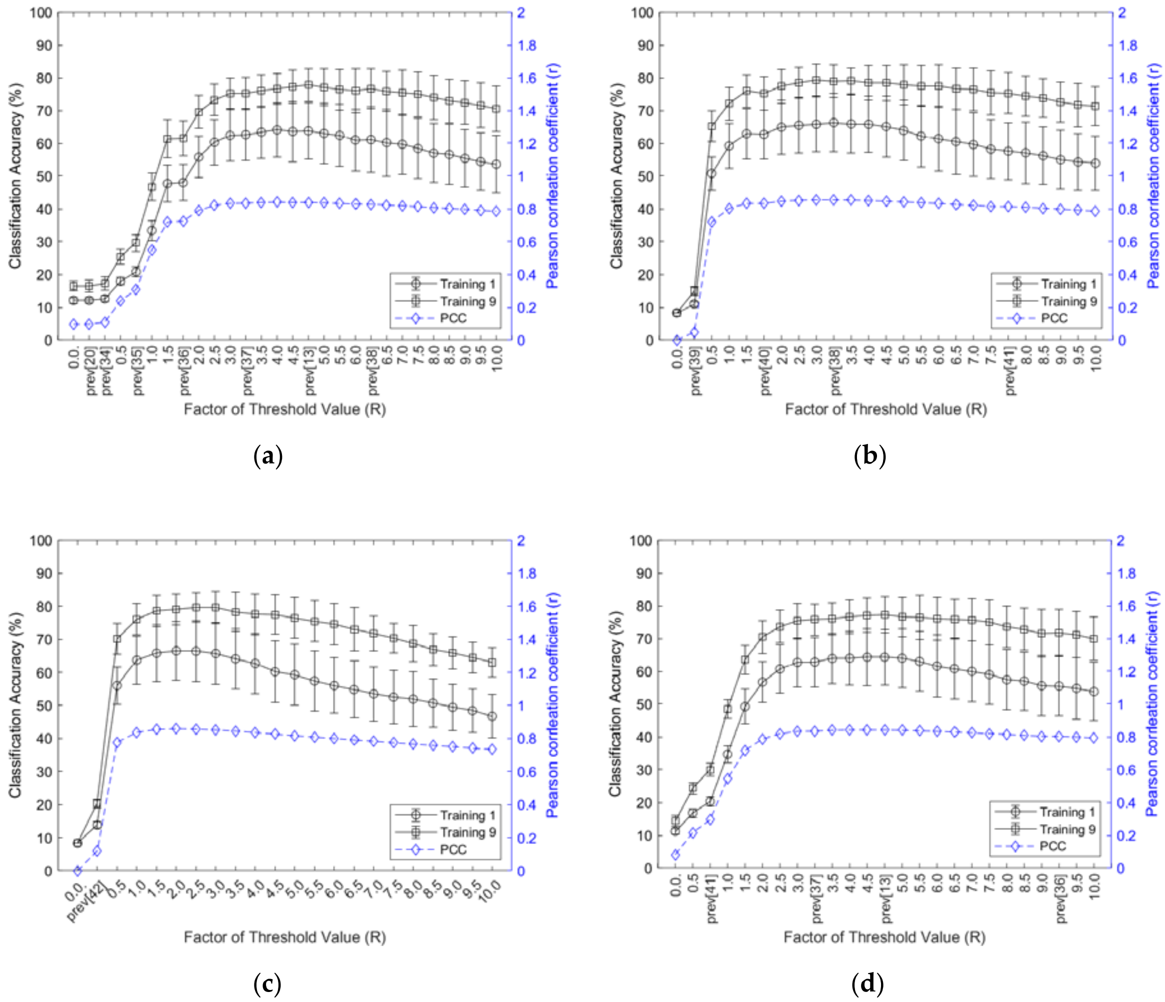

Figure S6. Classification accuracy and inter-session PCC of Group 7 by threshold values: (**a**) ZC; (**b**) WAMP; (**c**) MYOP; (**d**) SSC; prev: threshold value from the previous study.

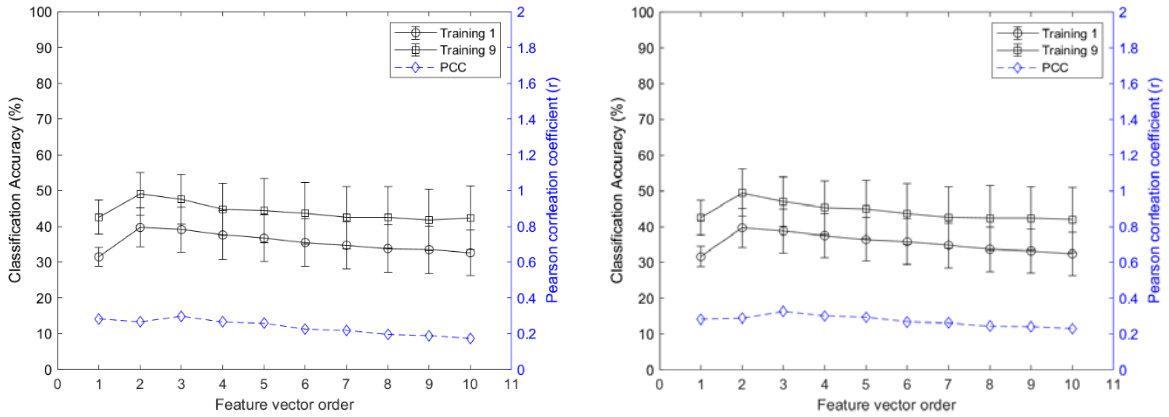

(**a**) (**b**)

Figure S7. Classification accuracy and inter-session PCC of Group 2 by feature vector order: (**a**) AR; (**b**) CC.

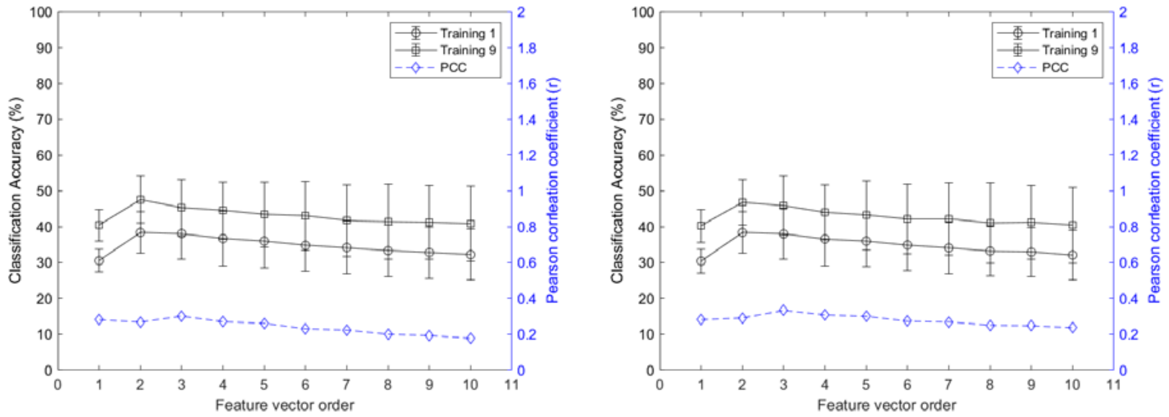

(**a**) (**b**)

Figure S8. Classification accuracy and inter-session PCC of Group 3 by feature vector order: (**a**) AR; (**b**) CC.

sensors

MDPI

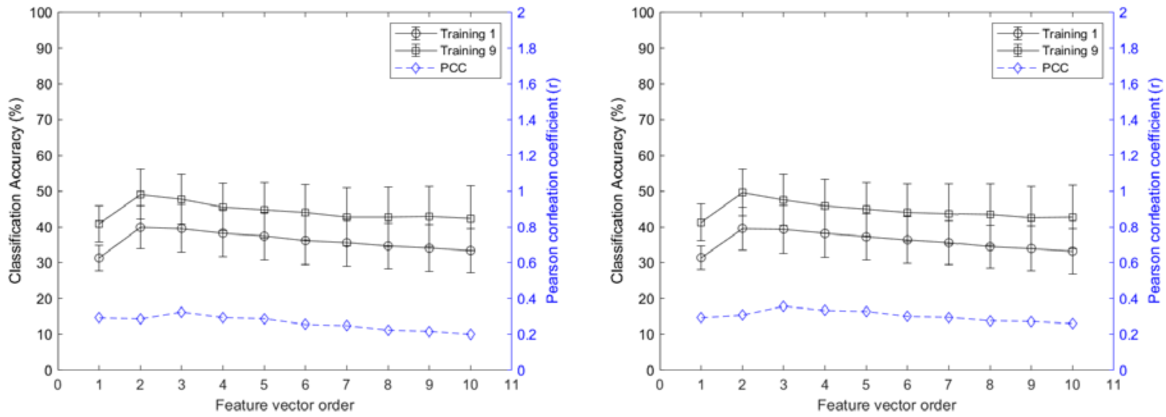

(**a**) (**b**)

Figure S9. Classification accuracy and inter-session PCC of Group 4 by feature vector order: (**a**) AR; (**b**) CC.

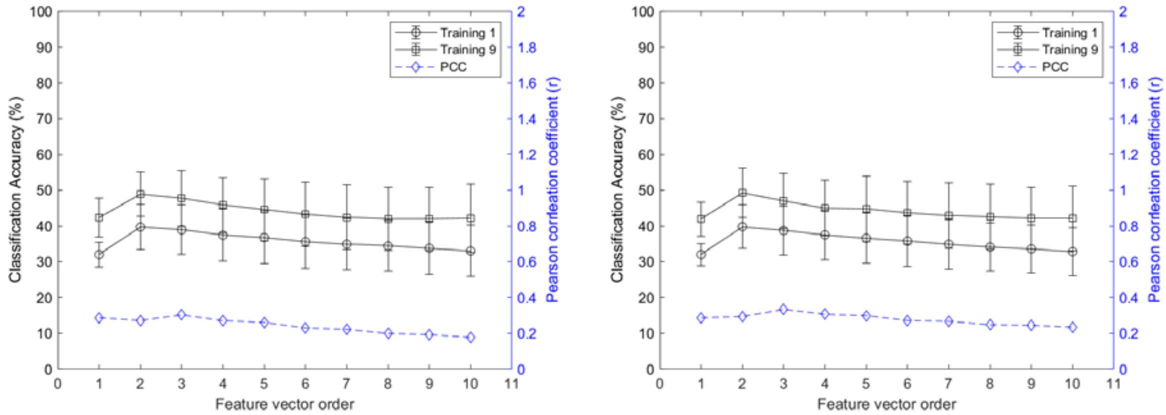

(**a**) (**b**)

Figure S10. Classification accuracy and inter-session PCC of Group 5 by feature vector order: (**a**) AR; (**b**) CC.

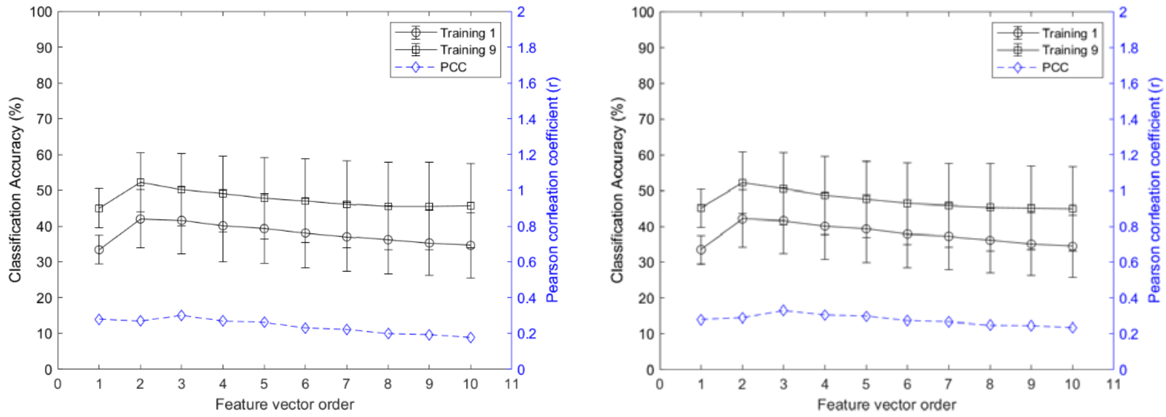

(**a**) (**b**)

Figure S11. Classification accuracy and inter-session PCC of Group 6 by feature vector order: (**a**) AR; (**b**) CC.

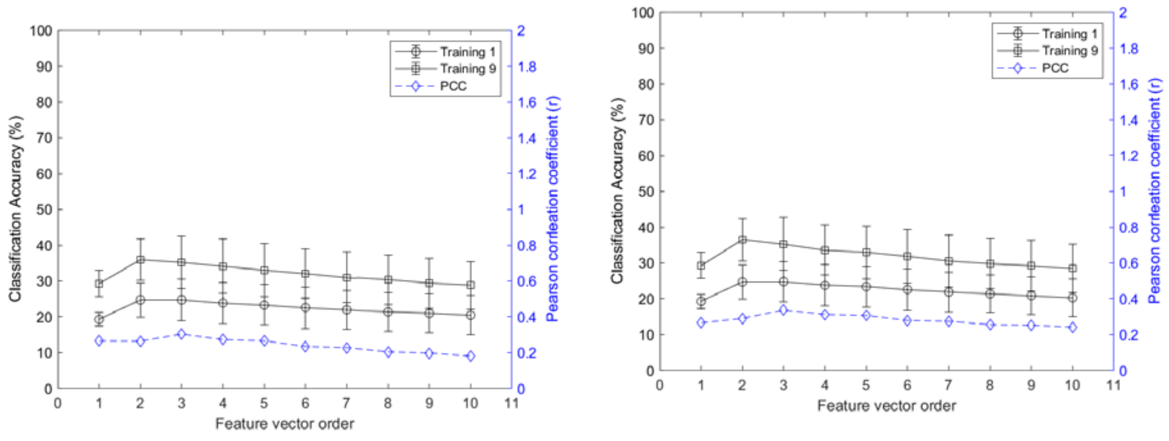

(**a**) (**b**)

Figure S12. Classification accuracy and inter-session PCC of Group 7 by feature vector order: (**a**) AR; (**b**) CC.

Table S1. Classification accuracy and inter-session PCC of the feature vectors in Group 2.

Feature vector	Classification accuracy (%): mean (standard deviation)									PCC (*r*)
	TRN1	TRN2	TRN3	TRN4	TRN5	TRN6	TRN7	TRN8	TRN9	
**IEMG**	80.8 (6.8)	87.2 (3.7)	89.4 (2.8)	90.9 (2.2)	91.3 (1.9)	91.8 (2.0)	92.1 (2.2)	92.7 (1.9)	93.2 (2.1)	0.831
**MAV**	80.9 (6.6)	87.0 (3.3)	89.8 (2.7)	90.8 (2.2)	91.4 (1.8)	91.9 (2.1)	92.3 (1.9)	92.4 (2.3)	93.6 (2.2)	0.831
**MAV1**	80.6 (6.6)	87.0 (3.6)	89.5 (2.5)	90.5 (2.0)	91.6 (1.6)	91.5 (2.0)	92.1 (2.2)	93.0 (1.8)	93.1 (1.9)	0.829
**MAV2**	79.5 (5.6)	85.1 (2.5)	86.8 (1.6)	88.1 (1.5)	88.5 (1.8)	89.2 (1.5)	89.8 (1.8)	90.2 (1.6)	90.6 (1.9)	0.802
**SSI**	74.9 (7.5)	83.2 (4.6)	87.1 (3.2)	88.4 (2.8)	89.8 (2.0)	90.5 (2.2)	91.1 (2.0)	91.5 (2.1)	92.6 (1.6)	0.735
**VAR**	75.7 (8.1)	82.8 (4.4)	86.5 (3.5)	88.3 (2.1)	89.6 (2.3)	90.5 (2.2)	91.5 (1.7)	92.0 (1.7)	91.7 (2.4)	0.735
**TM3**	61.6 (5.9)	70.2 (4.1)	73.6 (3.9)	75.7 (3.8)	76.8 (3.8)	77.5 (3.9)	78.3 (3.7)	78.5 (3.7)	78.8 (3.9)	0.393
**TM4**	64.8 (9.3)	74.3 (6.2)	77.8 (6.0)	80.2 (5.5)	81.7 (5.3)	82.4 (5.4)	83.6 (4.9)	83.7 (5.3)	84.7 (5.2)	0.430
**TM5**	57.1 (7.3)	63.8 (6.8)	65.8 (7.0)	66.3 (7.0)	66.8 (6.9)	66.9 (6.9)	66.8 (7.1)	67.0 (7.0)	67.2 (6.9)	0.249
**RMS**	80.1 (6.2)	86.8 (3.5)	89.4 (2.9)	90.8 (1.9)	91.4 (1.8)	91.7 (2.2)	92.7 (1.6)	92.6 (1.9)	93.1 (1.7)	0.822
**LOG**	81.3 (6.1)	85.9 (3.8)	88.3 (2.3)	89.3 (2.4)	89.8 (1.9)	90.4 (1.8)	90.8 (1.9)	90.9 (2.1)	91.4 (2.1)	0.849
**WL**	79.8 (7.1)	86.4 (3.6)	89.2 (2.6)	90.7 (2.0)	91.2 (1.8)	91.4 (2.1)	92.0 (1.7)	92.2 (1.7)	93.0 (1.8)	0.826
**AAC**	80.2 (6.6)	86.9 (3.3)	89.2 (2.4)	90.5 (2.2)	90.7 (1.7)	91.5 (1.6)	91.9 (1.7)	92.0 (2.0)	92.7 (1.6)	0.826
**DASDV**	79.9 (6.7)	86.6 (3.3)	89.1 (2.4)	90.3 (2.1)	91.3 (1.8)	91.7 (2.2)	91.5 (2.2)	92.3 (2.0)	92.2 (1.8)	0.817
**MAVSLP**	35.3 (3.2)	40.9 (3.3)	42.9 (3.2)	44.1 (3.1)	44.5 (3.2)	45.1 (3.0)	45.2 (3.0)	45.4 (3.1)	45.5 (3.1)	0.004
**ZC**	81.2 (6.6)	86.2 (3.3)	88.2 (2.6)	89.3 (2.5)	90.0 (2.0)	90.3 (2.2)	90.8 (2.1)	90.8 (2.3)	91.2 (2.3)	0.842
**WAMP**	82.6 (6.2)	87.6 (3.2)	89.8 (2.1)	90.7 (1.8)	91.2 (1.4)	91.3 (1.8)	91.6 (2.0)	91.9 (1.9)	92.7 (1.6)	0.857
**MYOP**	82.8 (5.9)	88.1 (3.1)	90.3 (1.9)	91.4 (1.6)	92.0 (1.8)	91.9 (1.8)	92.6 (1.9)	92.5 (2.2)	92.5 (2.2)	0.861

sensors
MDPI

**SSC**	81.3 (6.5)	86.6 (3.4)	88.7 (2.8)	89.0 (2.8)	89.9 (2.1)	90.5 (2.3)	90.7 (2.4)	91.1 (2.3)	91.1 (2.4)	0.844
**AR**	39.7 (5.4)	41.5 (6.4)	41.6 (6.7)	43.0 (6.4)	44.6 (6.0)	45.9 (5.3)	47.4 (5.7)	48.3 (5.8)	49.1 (6.0)	0.267
**CC**	39.7 (5.5)	41.5 (6.3)	41.8 (6.8)	43.2 (6.2)	44.5 (6.0)	45.9 (5.7)	47.1 (5.7)	47.7 (6.0)	49.5 (6.6)	0.288

Table S2. Classification accuracy and inter-session PCC of the feature vectors in Group 3.

Feature vector	Classification accuracy (%): mean (standard deviation)									PCC (*r*)
	TRN1	TRN2	TRN3	TRN4	TRN5	TRN6	TRN7	TRN8	TRN9	
**IEMG**	75.1 (7.2)	81.2 (4.9)	83.5 (4.6)	84.8 (4.0)	85.8 (4.1)	86.2 (3.9)	86.4 (3.8)	87.0 (3.4)	87.6 (3.2)	0.826
**MAV**	75.5 (7.9)	81.2 (4.8)	83.6 (4.3)	84.8 (4.2)	85.7 (4.2)	86.2 (3.5)	86.6 (3.5)	86.1 (3.7)	87.6 (3.6)	0.826
**MAV1**	75.4 (7.8)	81.2 (5.1)	83.0 (4.6)	84.4 (4.1)	85.3 (4.1)	86.0 (4.0)	86.2 (3.4)	86.8 (3.5)	86.4 (3.8)	0.824
**MAV2**	73.9 (7.4)	79.2 (5.5)	80.5 (5.6)	81.9 (5.3)	82.7 (5.2)	83.4 (4.6)	84.2 (4.2)	84.8 (4.4)	85.5 (3.9)	0.797
**SSI**	70.1 (8.1)	77.1 (4.7)	80.6 (3.8)	82.6 (2.8)	83.8 (3.3)	84.8 (2.7)	85.8 (2.6)	86.7 (2.6)	87.5 (2.9)	0.726
**VAR**	70.3 (8.2)	77.3 (4.8)	80.8 (3.6)	82.5 (3.3)	84.3 (3.0)	84.7 (3.2)	86.0 (2.5)	86.1 (3.0)	87.7 (2.3)	0.726
**TM3**	58.0 (6.2)	65.7 (5.4)	69.3 (5.1)	71.5 (4.6)	72.9 (5.0)	73.7 (5.0)	74.3 (5.2)	74.9 (5.2)	75.4 (5.5)	0.389
**TM4**	60.8 (6.9)	69.6 (5.3)	74.1 (4.2)	76.6 (2.9)	77.9 (3.2)	79.7 (3.4)	80.3 (3.3)	80.7 (3.6)	81.3 (3.9)	0.425
**TM5**	53.7 (5.9)	59.9 (6.0)	61.9 (6.0)	63.0 (5.8)	63.1 (6.0)	63.0 (6.0)	63.2 (6.1)	62.9 (6.3)	63.2 (6.5)	0.245
**RMS**	74.8 (7.1)	80.6 (5.0)	83.7 (4.1)	85.0 (3.7)	86.0 (3.8)	86.7 (3.6)	86.9 (3.7)	87.4 (2.7)	87.4 (3.4)	0.818
**LOG**	76.4 (6.9)	80.1 (5.3)	82.0 (5.5)	82.9 (5.4)	83.7 (5.3)	83.7 (5.5)	84.4 (5.2)	84.4 (5.2)	85.1 (5.2)	0.847
**WL**	74.9 (6.9)	80.8 (4.7)	83.2 (4.0)	84.4 (4.1)	85.3 (3.9)	86.0 (3.7)	86.2 (3.4)	86.8 (3.4)	86.9 (3.6)	0.822
**AAC**	74.9 (7.0)	80.6 (4.8)	83.3 (4.2)	84.5 (4.1)	85.6 (3.8)	86.1 (3.5)	86.4 (3.7)	87.0 (3.1)	86.6 (3.4)	0.822
**DASDV**	74.4 (7.2)	80.2 (4.8)	83.4 (4.1)	84.6 (3.8)	85.4 (3.6)	85.9 (3.7)	86.8 (3.4)	86.8 (3.3)	87.4 (3.3)	0.813
**MAVSLP**	34.4 (3.6)	39.5 (3.9)	41.4 (3.8)	42.5 (3.8)	43.0 (3.8)	43.3 (3.8)	43.6 (3.6)	44.0 (3.9)	44.0 (3.7)	0.005
**ZC**	76.1 (7.8)	79.9 (6.2)	82.2 (5.3)	82.7 (5.0)	83.0 (4.8)	83.8 (4.6)	84.3 (4.9)	84.4 (4.3)	85.7 (4.3)	0.845
**WAMP**	78.0 (7.2)	81.4 (6.2)	83.5 (5.4)	84.0 (5.0)	84.7 (5.1)	84.8 (5.0)	85.3 (4.9)	85.6 (4.8)	86.1 (4.4)	0.860
**MYOP**	78.4 (6.4)	81.9 (5.6)	83.5 (5.3)	84.5 (5.3)	85.3 (5.0)	85.1 (5.1)	85.8 (4.8)	85.8 (4.8)	86.7 (4.7)	0.864
**SSC**	76.5 (6.9)	80.1 (5.6)	81.5 (5.5)	82.7 (5.1)	82.9 (5.0)	83.5 (4.9)	83.9 (4.4)	84.2 (4.7)	84.6 (4.6)	0.849
**AR**	38.4 (5.8)	39.8 (6.7)	40.4 (6.9)	41.6 (6.6)	42.9 (6.2)	44.2 (6.0)	45.6 (6.2)	46.4 (6.4)	47.6 (6.6)	0.268
**CC**	38.4 (5.8)	40.0 (6.6)	40.2 (6.5)	41.5 (6.6)	43.1 (6.1)	44.2 (6.0)	45.2 (5.8)	46.3 (5.9)	46.8 (6.3)	0.291

sensors

Table S3. Classification accuracy and inter-session PCC of the feature vectors in Group 4.

Feature vector	Classification accuracy (%): mean (standard deviation)									PCC (*r*)
	**TRN1**	**TRN2**	**TRN3**	**TRN4**	**TRN5**	**TRN6**	**TRN7**	**TRN8**	**TRN9**	
**IEMG**	79.1 (7.6)	84.7 (5.2)	87.4 (4.4)	88.2 (4.3)	88.9 (4.0)	89.2 (4.0)	90.1 (3.9)	90.7 (4.0)	91.2 (4.0)	0.833
**MAV**	79.2 (7.8)	84.6 (5.1)	87.3 (4.6)	88.3 (4.2)	89.4 (3.8)	89.4 (3.8)	90.1 (3.8)	90.3 (4.2)	91.4 (3.6)	0.833
**MAV1**	79.6 (7.6)	84.4 (5.2)	86.9 (4.7)	88.1 (4.0)	88.8 (4.0)	89.5 (3.7)	90.2 (3.6)	90.4 (3.8)	91.4 (3.6)	0.831
**MAV2**	77.9 (7.6)	82.3 (5.2)	84.2 (4.8)	85.0 (4.3)	85.6 (4.3)	86.0 (4.4)	86.9 (4.4)	87.4 (4.1)	88.6 (4.0)	0.803
**SSI**	74.6 (8.9)	81.6 (5.8)	84.9 (5.4)	86.3 (4.5)	87.5 (4.1)	88.2 (4.1)	89.0 (4.0)	89.7 (4.0)	90.2 (4.1)	0.711
**VAR**	74.6 (8.5)	81.6 (5.7)	84.9 (5.0)	86.2 (4.7)	87.5 (4.5)	88.3 (3.9)	89.4 (3.9)	89.8 (4.3)	89.9 (4.2)	0.711
**TM3**	61.0 (7.1)	67.9 (6.0)	71.0 (5.3)	73.3 (5.2)	74.4 (5.1)	75.7 (5.2)	76.1 (5.1)	76.3 (5.7)	76.8 (5.8)	0.364
**TM4**	65.8 (9.4)	73.7 (6.8)	77.8 (6.2)	79.9 (5.3)	81.0 (5.1)	81.8 (5.2)	82.7 (5.2)	83.4 (5.0)	84.4 (4.9)	0.381
**TM5**	56.6 (7.5)	62.2 (6.6)	64.2 (6.7)	65.0 (6.8)	65.1 (7.0)	65.1 (7.5)	64.9 (7.7)	64.9 (7.9)	64.7 (8.4)	0.207
**RMS**	79.2 (7.9)	84.6 (5.5)	87.5 (4.5)	88.5 (4.2)	89.3 (4.0)	89.9 (3.7)	90.2 (3.9)	90.7 (3.9)	91.5 (3.5)	0.824
**LOG**	80.4 (7.2)	84.4 (5.0)	86.5 (4.0)	87.5 (3.5)	87.9 (3.8)	88.3 (3.9)	88.4 (3.8)	88.9 (3.5)	89.1 (3.7)	0.863
**WL**	78.7 (7.6)	84.7 (5.2)	86.7 (4.9)	88.0 (4.1)	89.0 (3.9)	89.3 (3.9)	89.3 (4.0)	89.8 (4.0)	90.5 (4.1)	0.827
**AAC**	78.7 (7.8)	84.6 (5.4)	86.8 (4.8)	88.1 (4.1)	88.6 (3.9)	89.0 (4.1)	89.2 (4.1)	89.3 (4.3)	90.3 (4.0)	0.827
**DASDV**	78.2 (8.3)	84.2 (5.6)	86.9 (4.9)	87.8 (4.4)	88.6 (4.3)	88.9 (4.3)	89.2 (4.4)	89.8 (4.2)	90.2 (4.3)	0.819
**MAVSLP**	36.0 (3.3)	41.1 (3.4)	43.1 (3.3)	44.0 (3.3)	44.5 (3.3)	45.0 (3.3)	45.2 (2.9)	45.5 (3.4)	45.3 (3.2)	0.005
**ZC**	78.4 (7.3)	82.6 (5.8)	84.2 (5.0)	85.1 (4.0)	85.3 (4.3)	85.8 (4.4)	85.9 (4.4)	85.8 (4.8)	86.6 (4.6)	0.868
**WAMP**	80.1 (7.1)	85.0 (5.1)	86.6 (4.6)	87.3 (4.3)	88.1 (3.9)	88.5 (3.7)	88.6 (4.0)	89.3 (3.6)	89.5 (3.7)	0.883
**MYOP**	80.4 (7.5)	85.4 (5.0)	87.1 (4.1)	87.9 (3.8)	88.7 (3.7)	88.7 (3.8)	89.4 (4.0)	88.9 (4.5)	89.8 (3.8)	0.886
**SSC**	77.9 (7.6)	82.3 (5.6)	84.3 (5.0)	85.2 (4.5)	85.6 (4.7)	85.9 (4.7)	85.7 (4.7)	86.2 (5.3)	86.9 (5.0)	0.872
**AR**	39.9 (6.0)	41.7 (6.6)	42.2 (7.1)	42.8 (6.5)	44.5 (6.1)	46.1 (6.1)	47.6 (6.2)	48.1 (6.9)	49.1 (7.0)	0.286
**CC**	39.6 (6.0)	41.8 (6.6)	41.9 (6.7)	43.3 (6.2)	44.3 (6.0)	45.9 (5.8)	47.1 (6.2)	48.4 (6.5)	49.6 (6.5)	0.307

Table S4. Classification accuracy and inter-session PCC of the feature vectors in Group 5.

Feature vector	Classification accuracy (%): mean (standard deviation)									PCC (*r*)
	TRN1	TRN2	TRN3	TRN4	TRN5	TRN6	TRN7	TRN8	TRN9	
**IEMG**	79.7 (6.4)	84.9 (4.8)	87.5 (3.5)	89.1 (3.3)	89.3 (3.2)	89.8 (2.6)	90.7 (2.4)	90.8 (3.2)	91.3 (3.1)	0.829
**MAV**	79.5 (6.4)	85.0 (4.8)	88.1 (3.3)	88.6 (3.5)	89.9 (2.9)	90.1 (2.2)	90.7 (2.4)	90.8 (2.7)	91.3 (3.7)	0.829
**MAV1**	79.6 (6.6)	85.1 (4.7)	87.6 (3.5)	88.6 (3.4)	89.5 (3.1)	90.0 (2.4)	90.3 (2.2)	90.6 (3.2)	90.7 (4.3)	0.827
**MAV2**	78.3 (5.7)	83.0 (4.2)	84.9 (3.2)	86.2 (2.7)	86.4 (2.4)	87.3 (2.1)	87.9 (2.2)	88.7 (2.2)	89.2 (2.0)	0.800
**SSI**	74.5 (7.2)	81.2 (5.2)	84.5 (4.3)	87.0 (3.4)	87.9 (3.2)	88.8 (2.7)	89.4 (2.9)	90.4 (2.6)	91.1 (3.0)	0.730
**VAR**	74.4 (7.6)	80.9 (5.1)	84.6 (4.6)	86.6 (3.1)	87.9 (3.1)	88.7 (2.9)	90.0 (3.0)	90.4 (2.4)	90.7 (3.7)	0.730
**TM3**	60.6 (5.7)	68.4 (4.1)	72.0 (3.6)	74.2 (3.3)	75.6 (3.6)	76.5 (3.4)	77.2 (3.6)	77.9 (3.7)	77.7 (3.9)	0.392
**TM4**	64.6 (8.1)	72.8 (6.9)	76.8 (5.6)	79.4 (5.2)	80.9 (4.9)	81.9 (5.1)	82.9 (4.8)	83.5 (5.3)	84.1 (5.3)	0.428
**TM5**	56.5 (7.2)	63.2 (6.3)	65.0 (6.7)	65.7 (6.7)	65.6 (7.1)	65.5 (7.4)	65.9 (7.2)	65.8 (7.6)	66.0 (7.5)	0.248
**RMS**	79.4 (6.7)	85.1 (4.5)	87.7 (3.5)	89.2 (2.8)	89.5 (3.1)	89.9 (2.3)	90.6 (2.3)	90.7 (2.8)	91.2 (3.4)	0.820
**LOG**	80.2 (5.9)	84.6 (4.6)	86.3 (4.4)	88.0 (3.8)	88.1 (3.6)	88.6 (3.8)	88.9 (3.3)	89.0 (3.4)	88.9 (4.2)	0.849
**WL**	79.0 (6.9)	84.7 (4.6)	87.5 (3.9)	88.7 (3.2)	89.4 (3.1)	89.6 (2.6)	90.4 (2.7)	90.8 (2.8)	91.3 (3.2)	0.824
**AAC**	79.1 (6.9)	85.0 (4.8)	87.2 (4.1)	88.4 (3.2)	89.2 (3.0)	89.7 (2.7)	90.4 (2.6)	90.7 (3.3)	91.5 (3.5)	0.824
**DASDV**	79.0 (7.2)	84.6 (4.6)	87.2 (3.9)	88.2 (3.5)	89.4 (3.0)	89.9 (2.9)	90.4 (2.9)	90.7 (2.9)	91.5 (3.2)	0.815
**MAVSLP**	35.3 (3.3)	40.6 (3.4)	42.6 (3.7)	43.7 (3.5)	44.2 (3.5)	44.6 (3.6)	44.9 (3.6)	45.1 (3.6)	45.1 (3.7)	0.005
**ZC**	79.9 (6.3)	84.3 (4.1)	86.0 (4.1)	87.2 (3.7)	88.0 (3.4)	88.5 (3.2)	88.6 (2.8)	89.1 (3.0)	89.4 (2.9)	0.845
**WAMP**	81.3 (6.0)	85.8 (4.2)	88.2 (3.7)	88.9 (3.6)	89.2 (3.5)	89.6 (3.4)	89.9 (3.5)	90.1 (3.3)	90.8 (3.2)	0.860
**MYOP**	81.2 (5.5)	85.9 (3.7)	88.1 (3.6)	89.1 (2.9)	89.8 (2.7)	90.3 (2.6)	90.2 (2.5)	90.6 (2.4)	90.7 (3.0)	0.864
**SSC**	80.1 (5.4)	84.3 (4.1)	86.3 (3.6)	87.4 (3.2)	87.7 (3.2)	88.2 (2.8)	88.6 (2.8)	88.6 (3.0)	88.9 (2.4)	0.849
**AR**	39.7 (6.3)	41.5 (7.1)	41.7 (7.5)	43.2 (6.9)	44.6 (6.6)	46.1 (6.4)	47.4 (6.2)	48.4 (6.6)	48.9 (6.2)	0.272
**CC**	39.8 (6.0)	41.7 (7.1)	41.9 (7.3)	43.2 (6.9)	44.8 (6.5)	46.0 (6.2)	47.4 (6.5)	48.3 (6.6)	49.2 (6.9)	0.294

Table S5. Classification accuracy and inter-session PCC of the feature vectors in Group 6.

Feature vector	Classification accuracy (%): mean (standard deviation)									PCC (*r*)
	TRN1	TRN2	TRN3	TRN4	TRN5	TRN6	TRN7	TRN8	TRN9	
**IEMG**	83.7 (8.2)	89.3 (5.4)	91.4 (4.3)	92.8 (3.7)	93.6 (3.3)	94.2 (2.9)	94.5 (2.8)	94.7 (2.8)	95.3 (2.3)	0.823
**MAV**	83.6 (8.6)	89.1 (5.7)	91.4 (4.3)	92.6 (3.6)	93.4 (3.4)	94.4 (3.1)	94.7 (2.7)	94.7 (2.9)	95.5 (2.0)	0.823
**MAV1**	83.9 (8.2)	89.2 (5.6)	91.1 (4.3)	92.6 (3.7)	93.4 (3.4)	94.4 (2.8)	94.2 (2.8)	94.6 (2.7)	95.3 (2.2)	0.821
**MAV2**	82.7 (8.2)	87.6 (5.8)	88.9 (5.2)	90.1 (4.7)	90.9 (4.2)	91.9 (4.0)	92.5 (3.9)	92.7 (3.5)	93.2 (3.4)	0.794
**SSI**	78.2 (8.8)	85.6 (6.5)	88.4 (5.5)	90.4 (4.4)	91.4 (4.0)	92.5 (3.5)	93.0 (3.2)	93.8 (2.8)	93.5 (2.8)	0.725
**VAR**	78.4 (8.9)	85.7 (6.2)	88.5 (5.4)	90.5 (4.3)	91.5 (4.0)	92.8 (3.1)	93.2 (3.1)	93.6 (3.1)	93.7 (3.2)	0.725
**TM3**	64.2 (6.8)	72.5 (5.8)	75.6 (5.7)	77.1 (5.6)	78.3 (5.8)	78.7 (6.1)	78.9 (6.5)	79.1 (6.9)	79.3 (7.5)	0.394
**TM4**	68.0 (9.2)	76.0 (9.2)	79.9 (8.0)	81.4 (7.4)	82.6 (7.5)	83.0 (7.7)	83.6 (7.8)	83.6 (8.2)	83.7 (8.8)	0.425
**TM5**	58.8 (8.8)	64.2 (9.2)	65.6 (9.4)	66.3 (9.7)	66.3 (10.0)	66.3 (10.6)	66.1 (10.8)	65.8 (11.0)	65.3 (11.7)	0.253
**RMS**	83.2 (8.2)	89.0 (5.4)	90.9 (4.6)	92.4 (3.9)	93.5 (3.3)	94.3 (3.1)	94.4 (3.0)	94.6 (2.8)	95.3 (2.4)	0.814
**LOG**	84.6 (7.1)	88.4 (5.6)	90.0 (4.7)	91.0 (4.1)	91.8 (4.2)	92.3 (4.0)	92.4 (4.0)	92.8 (4.0)	93.2 (3.6)	0.843
**WL**	83.1 (8.6)	88.8 (5.8)	91.1 (4.3)	92.3 (3.8)	93.2 (3.3)	93.9 (3.4)	94.3 (3.2)	93.9 (3.3)	94.9 (2.8)	0.818
**AAC**	83.6 (8.4)	88.9 (5.6)	90.8 (4.6)	92.2 (3.8)	93.0 (3.4)	93.8 (3.3)	94.0 (3.0)	94.3 (2.9)	94.8 (3.1)	0.818
**DASDV**	82.7 (9.1)	88.6 (5.8)	90.9 (4.5)	92.4 (3.5)	93.3 (3.2)	94.0 (3.0)	94.1 (3.1)	94.4 (2.7)	94.3 (2.7)	0.810
**MAVSLP**	37.6 (4.4)	42.9 (4.6)	45.0 (4.7)	45.8 (4.8)	46.4 (4.6)	46.9 (4.8)	47.1 (4.7)	47.2 (4.9)	47.4 (4.5)	0.005
**ZC**	84.3 (6.9)	87.9 (4.9)	89.7 (4.5)	91.1 (4.0)	91.9 (4.0)	92.4 (3.8)	92.5 (3.9)	93.1 (3.7)	93.1 (3.8)	0.840
**WAMP**	85.8 (6.5)	89.5 (4.7)	91.1 (4.2)	92.3 (3.9)	93.1 (3.7)	93.6 (3.6)	93.8 (3.9)	94.3 (3.5)	94.5 (3.5)	0.854
**MYOP**	86.2 (6.4)	90.2 (4.4)	91.7 (3.7)	92.8 (3.4)	93.6 (3.0)	93.9 (3.1)	94.2 (3.1)	94.4 (3.1)	94.7 (3.1)	0.858
**SSC**	84.7 (6.3)	88.5 (4.8)	89.9 (4.2)	91.5 (3.8)	92.0 (3.7)	92.7 (3.5)	92.7 (3.5)	93.1 (3.5)	92.9 (3.7)	0.843
**AR**	42.0 (8.1)	43.9 (9.4)	44.8 (9.4)	45.9 (9.5)	47.5 (8.9)	48.5 (8.5)	50.0 (8.4)	51.1 (8.2)	52.2 (8.2)	0.270
**CC**	42.1 (8.0)	44.2 (9.3)	44.7 (9.5)	46.0 (9.2)	47.0 (8.9)	48.8 (8.8)	49.9 (8.6)	50.9 (7.9)	52.2 (8.6)	0.290

sensors

MDPI

Table S6. Classification accuracy and inter-session PCC of the feature vectors in Group 7.

Feature vector	Classification accuracy (%): mean (standard deviation)									PCC (r)
	TRN1	TRN2	TRN3	TRN4	TRN5	TRN6	TRN7	TRN8	TRN9	
**IEMG**	65.6 (10.4)	71.8 (7.6)	74.9 (6.0)	76.2 (5.8)	77.4 (5.6)	78.4 (5.2)	78.9 (5.1)	80.1 (5.3)	81.0 (4.8)	0.821
**MAV**	65.5 (10.2)	71.8 (7.6)	74.5 (6.6)	76.4 (5.9)	77.2 (5.5)	78.5 (5.0)	78.8 (5.3)	79.8 (5.0)	80.9 (5.2)	0.821
**MAV1**	65.5 (10.6)	71.7 (7.3)	74.2 (6.3)	76.1 (5.5)	77.0 (5.4)	78.4 (5.0)	78.7 (5.0)	79.4 (5.2)	80.1 (5.5)	0.819
**MAV2**	63.1 (8.7)	68.3 (6.9)	71.3 (6.1)	73.6 (5.4)	75.3 (5.0)	76.8 (4.7)	77.8 (4.4)	78.9 (4.4)	79.6 (4.4)	0.792
**SSI**	61.2 (10.5)	69.0 (8.0)	72.9 (6.3)	75.6 (5.6)	77.4 (5.1)	79.0 (5.1)	79.9 (4.7)	80.3 (5.0)	81.6 (5.2)	0.709
**VAR**	61.3 (11.0)	68.7 (8.0)	73.2 (6.4)	75.5 (6.1)	77.3 (5.1)	79.1 (4.9)	80.0 (4.9)	81.1 (4.8)	81.6 (5.3)	0.709
**TM3**	48.0 (6.1)	54.5 (5.7)	57.1 (5.8)	58.6 (5.6)	59.4 (6.0)	59.8 (6.5)	60.4 (6.9)	60.2 (7.4)	60.8 (8.0)	0.379
**TM4**	52.9 (9.3)	61.0 (8.6)	64.3 (8.4)	65.6 (8.5)	66.6 (8.8)	67.4 (9.1)	67.9 (9.7)	68.0 (10.0)	67.6 (11.2)	0.395
**TM5**	42.6 (6.9)	46.3 (7.1)	46.9 (7.2)	46.7 (7.6)	46.5 (7.3)	46.2 (7.5)	46.2 (8.4)	45.9 (8.8)	46.0 (9.0)	0.229
**RMS**	65.1 (10.1)	71.8 (7.6)	74.7 (6.6)	76.2 (5.4)	77.4 (5.3)	78.8 (4.9)	79.3 (5.3)	80.3 (5.1)	81.0 (5.3)	0.811
**LOG**	65.4 (9.4)	70.0 (6.9)	71.4 (6.0)	72.8 (5.6)	73.8 (5.2)	75.1 (5.0)	75.8 (5.0)	77.0 (5.0)	78.2 (5.0)	0.844
**WL**	64.7 (9.9)	71.3 (7.1)	73.8 (6.2)	75.3 (5.8)	76.8 (5.2)	77.3 (5.1)	78.6 (4.7)	79.1 (5.0)	80.7 (4.8)	0.816
**AAC**	64.9 (10.1)	71.4 (7.1)	73.6 (6.4)	75.4 (5.5)	76.6 (5.7)	77.6 (5.0)	78.3 (5.1)	79.1 (5.0)	81.0 (4.5)	0.816
**DASDV**	63.9 (10.7)	70.7 (7.5)	74.0 (6.3)	75.6 (5.3)	77.1 (5.1)	78.0 (4.9)	79.0 (5.0)	79.7 (5.1)	80.6 (4.9)	0.807
**MAVSLP**	25.4 (2.5)	28.2 (2.5)	29.4 (2.5)	29.9 (2.7)	30.3 (2.3)	30.4 (2.6)	30.5 (2.4)	30.7 (2.5)	30.9 (2.8)	0.006
**ZC**	64.1 (8.2)	67.8 (6.8)	69.9 (5.5)	70.9 (5.0)	72.5 (4.8)	73.3 (4.9)	74.8 (4.8)	75.8 (5.1)	76.7 (4.7)	0.841
**WAMP**	65.8 (8.4)	71.0 (7.0)	72.4 (5.9)	73.4 (5.6)	75.0 (5.0)	75.8 (5.0)	76.9 (5.0)	77.9 (4.6)	79.3 (4.9)	0.855
**MYOP**	66.5 (9.0)	71.5 (7.2)	73.2 (6.1)	74.5 (5.5)	75.3 (5.0)	76.7 (4.9)	77.1 (5.1)	78.2 (5.0)	79.1 (4.7)	0.859
**SSC**	64.0 (8.1)	68.2 (6.8)	70.1 (5.8)	71.3 (5.2)	72.3 (5.0)	73.5 (4.9)	74.8 (4.7)	75.4 (5.5)	76.6 (5.1)	0.843
**AR**	24.7 (4.7)	26.5 (5.1)	29.0 (5.1)	31.0 (5.3)	32.4 (5.4)	33.6 (5.6)	34.8 (5.7)	35.6 (5.7)	35.9 (5.8)	0.265
**CC**	24.7 (4.7)	26.6 (5.0)	29.1 (5.1)	31.0 (5.3)	32.3 (5.5)	33.4 (5.5)	34.6 (5.7)	35.6 (5.6)	36.5 (5.9)	0.291

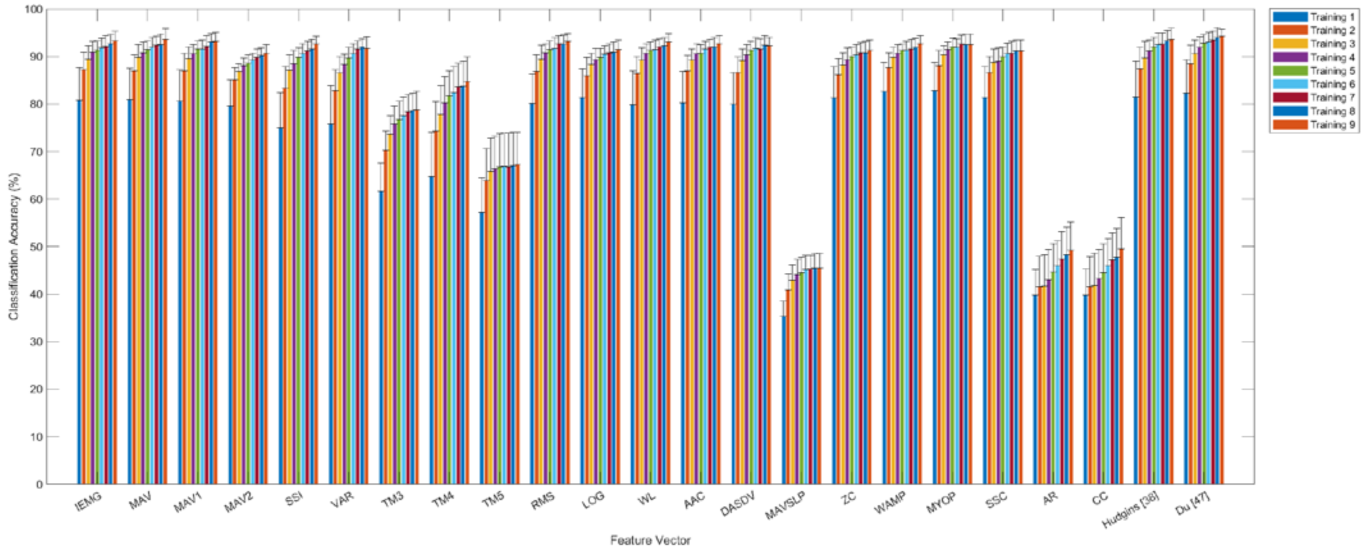

Figure S13. Classification accuracy of feature vectors and combinations by number of training sessions in Group 2.

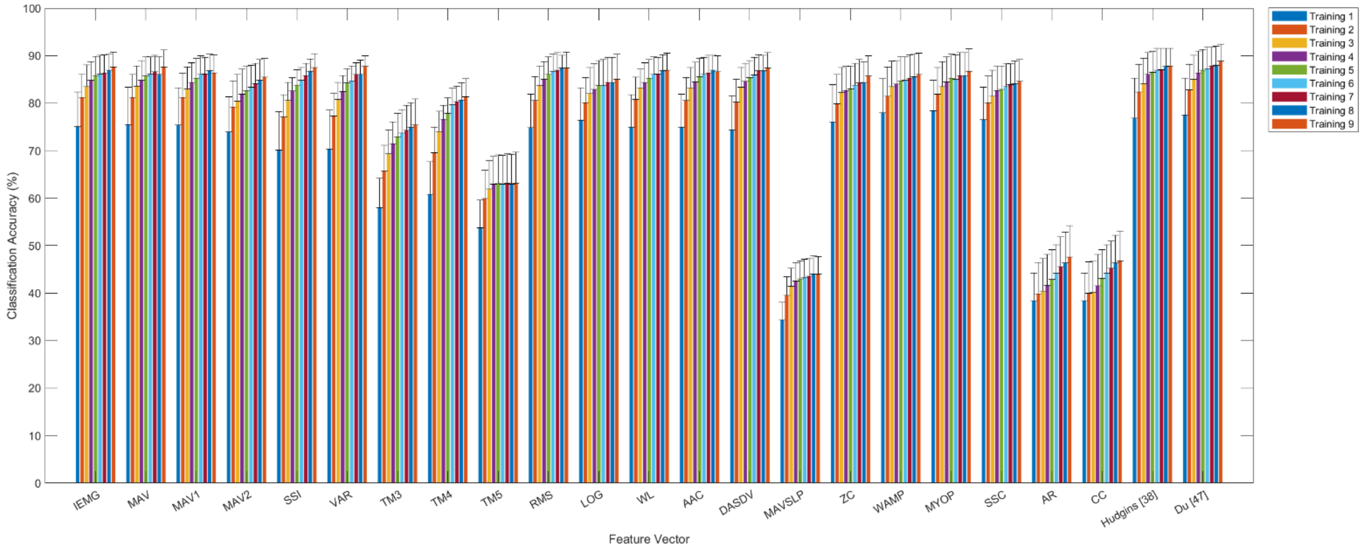

Figure S14. Classification accuracy of feature vectors and combinations by number of training sessions in Group 3.

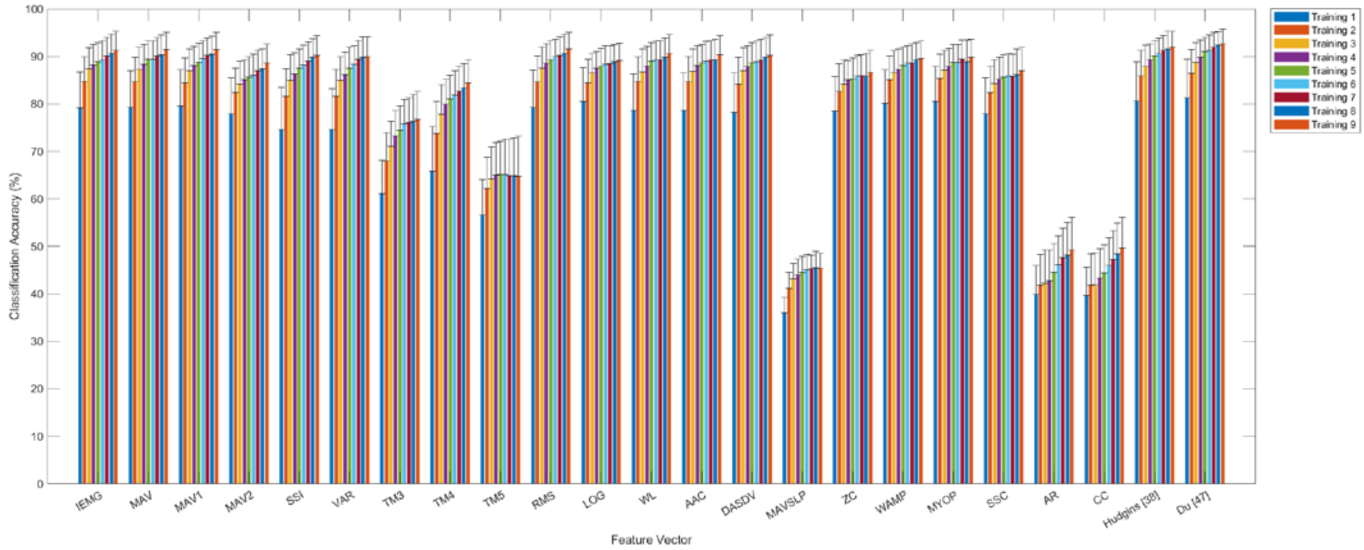

Figure S15. Classification accuracy of feature vectors and combinations by number of training sessions in Group 4.

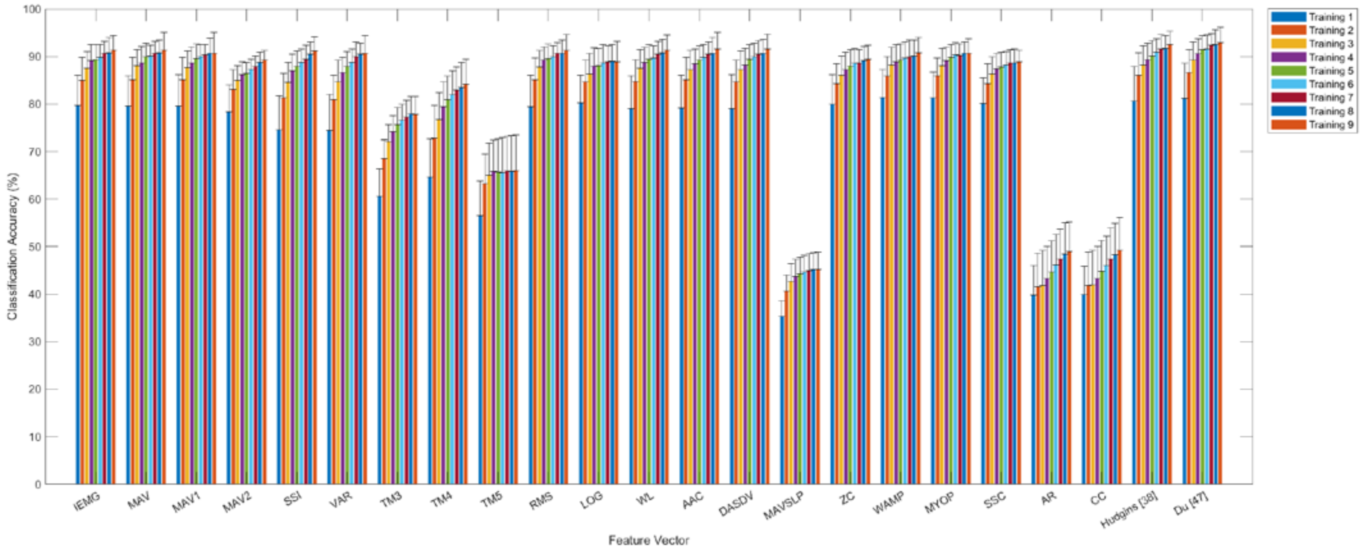

Figure S16. Classification accuracy of feature vectors and combinations by number of training sessions in Group 5

sensors MDPI

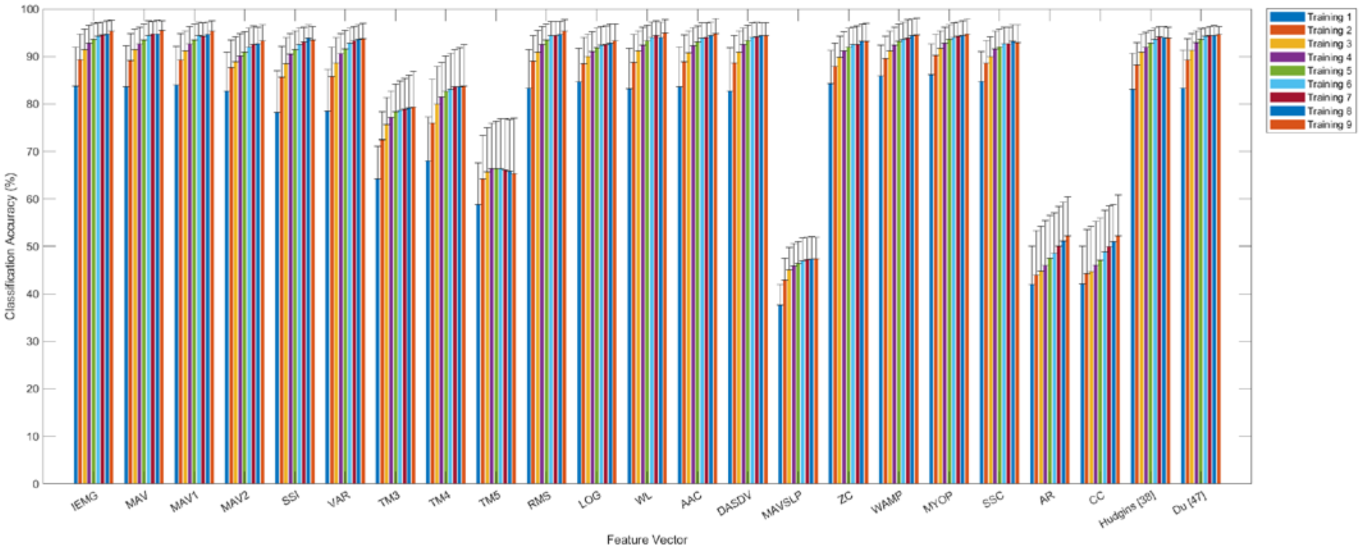

Figure S17. Classification accuracy of feature vectors and combinations by number of training sessions in Group 6.

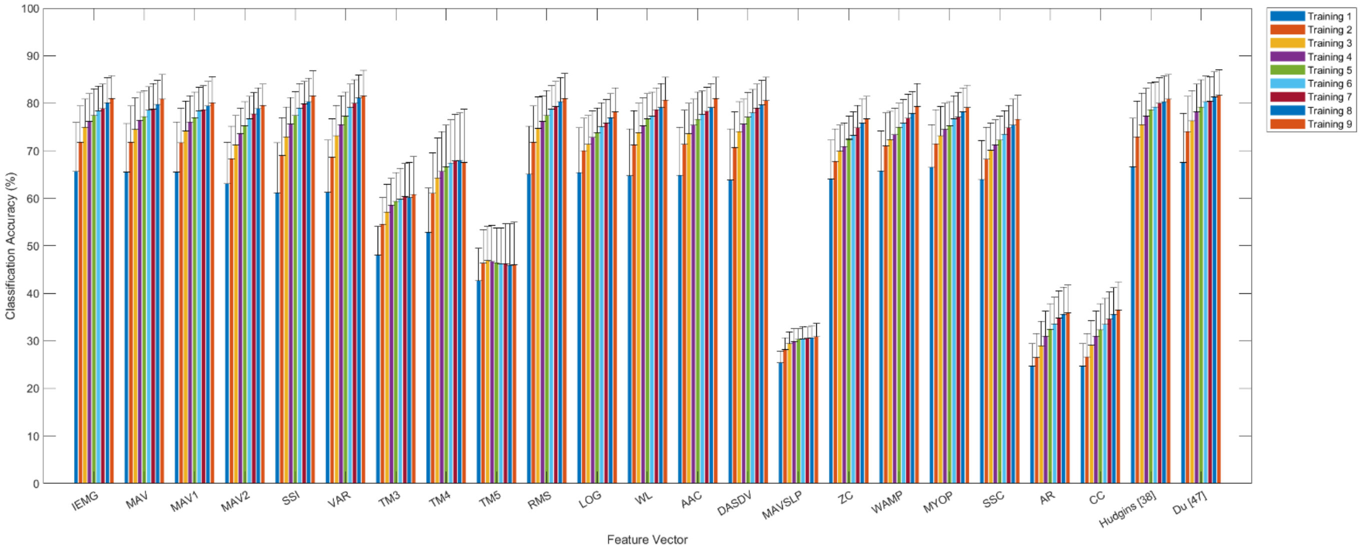

Figure S18. Classification accuracy of feature vectors and combinations by number of training sessions in Group 7.

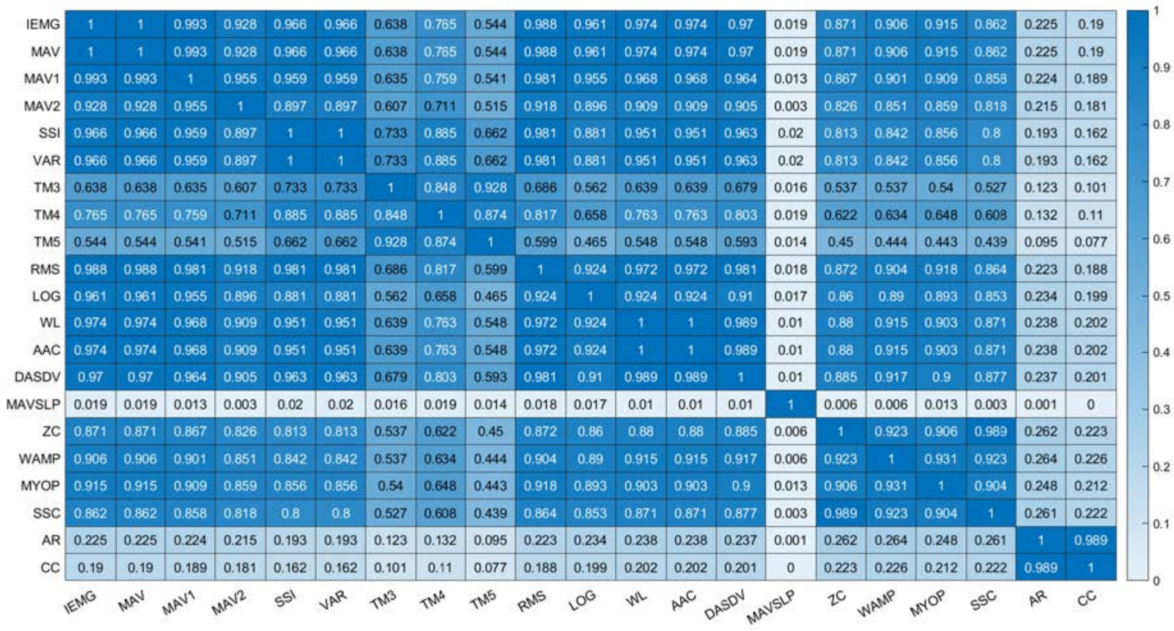

Figure S19. Confusion matrix of inter-feature PCC in Group 2.

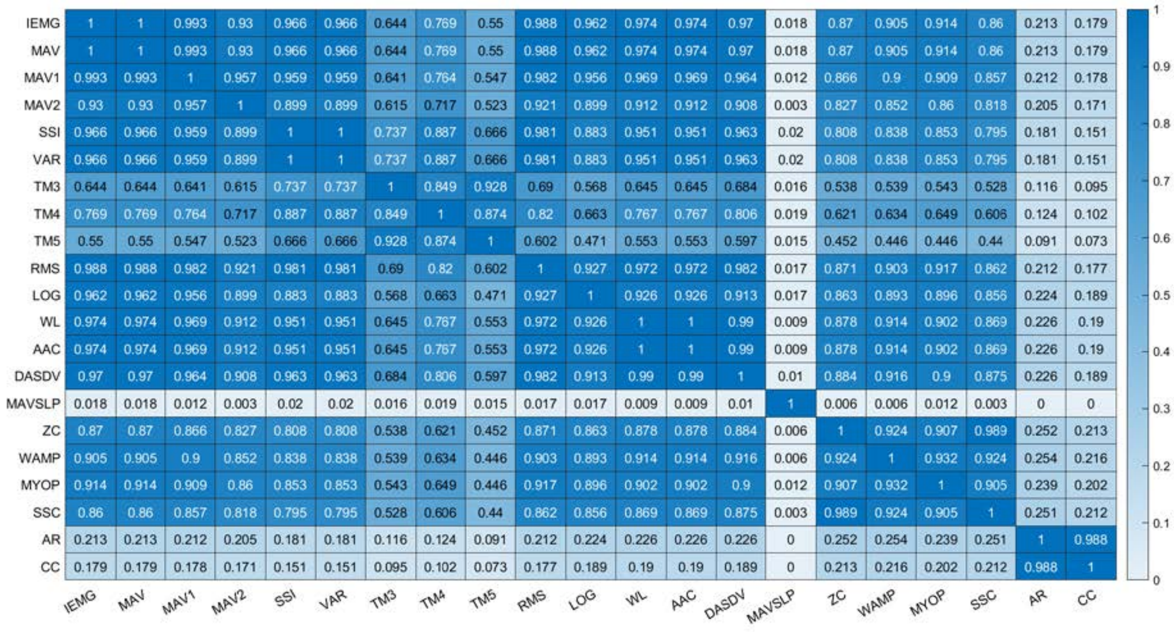

Figure S20. Confusion matrix of inter-feature PCC in Group 3.

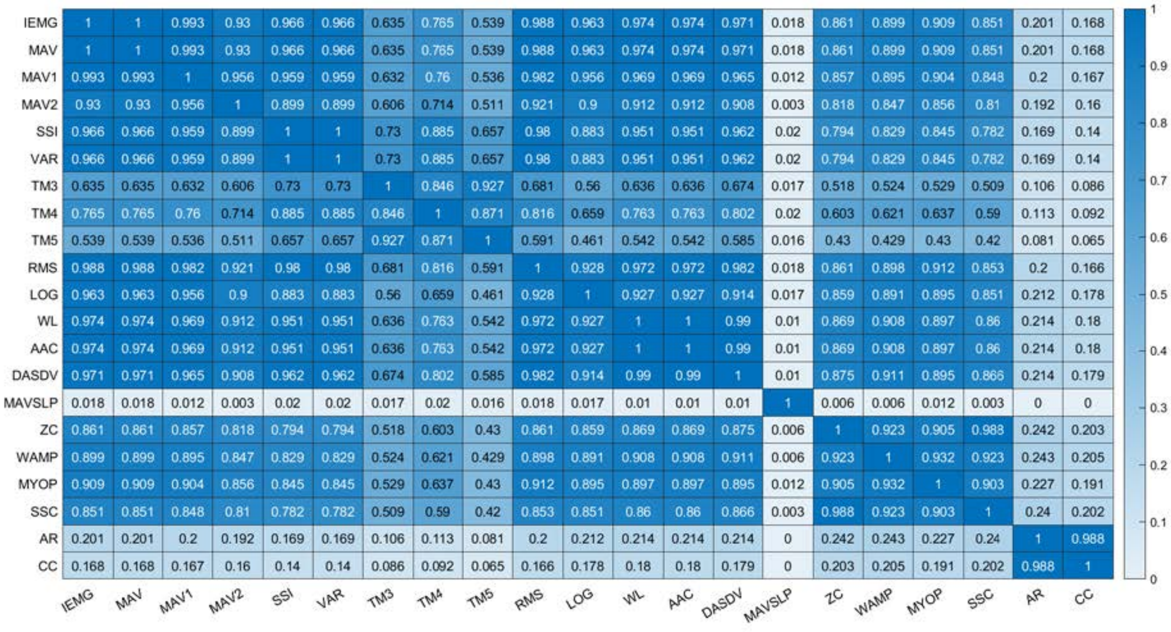

Figure S21. Confusion matrix of inter-feature PCC in Group 4.

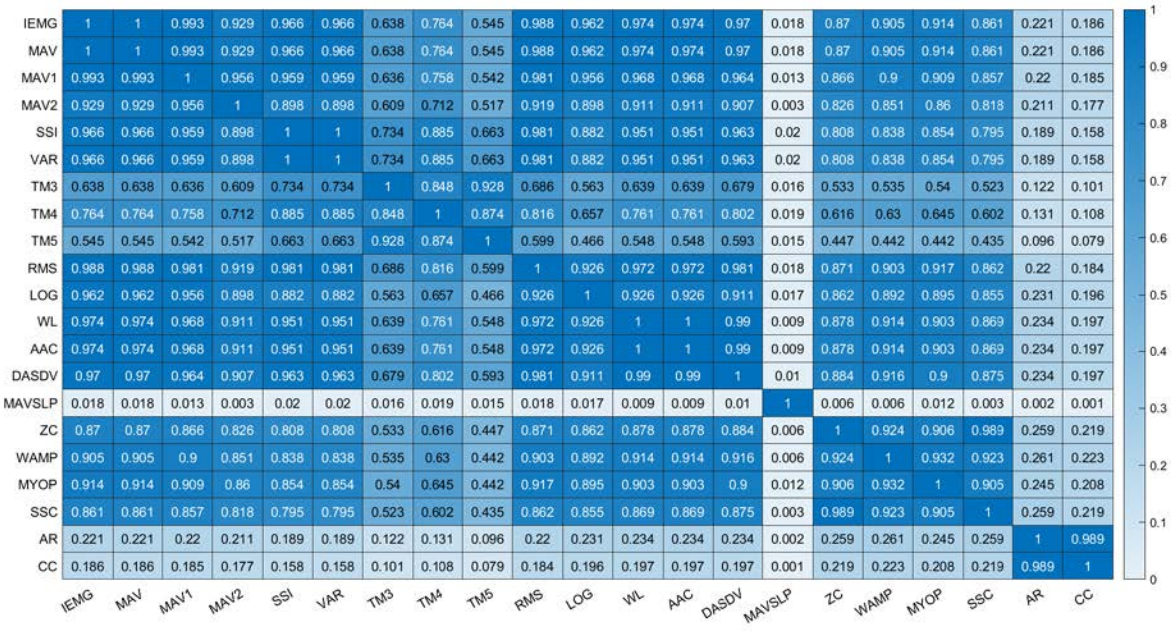

Figure S22. Confusion matrix of inter-feature PCC in Group 5.

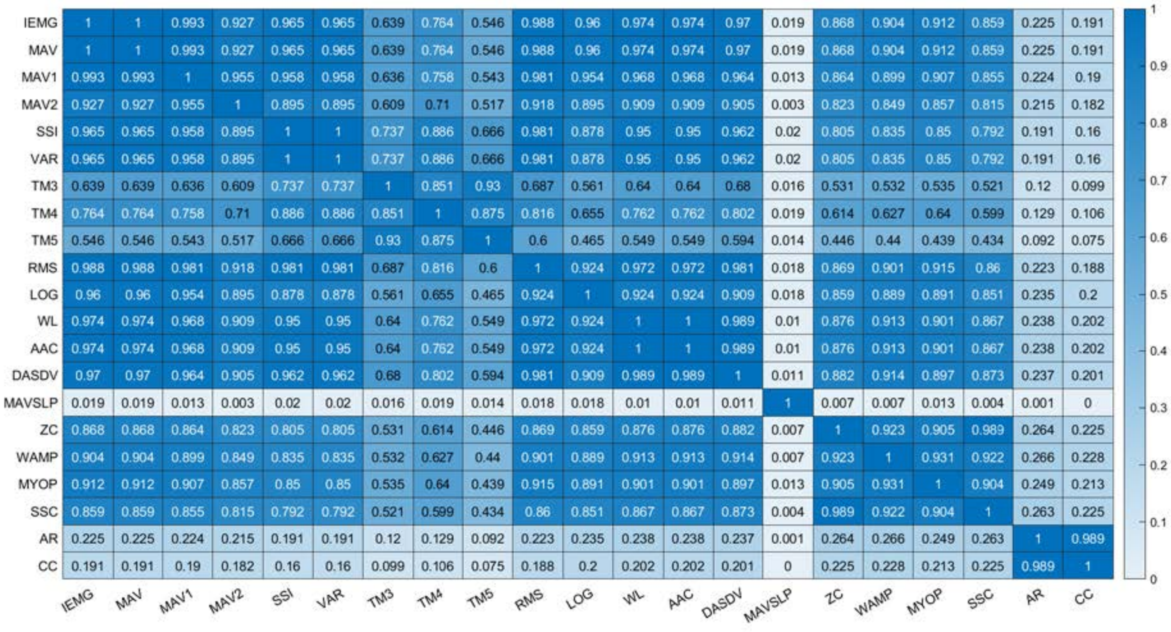

Figure S23. Confusion matrix of inter-feature PCC in Group 6.

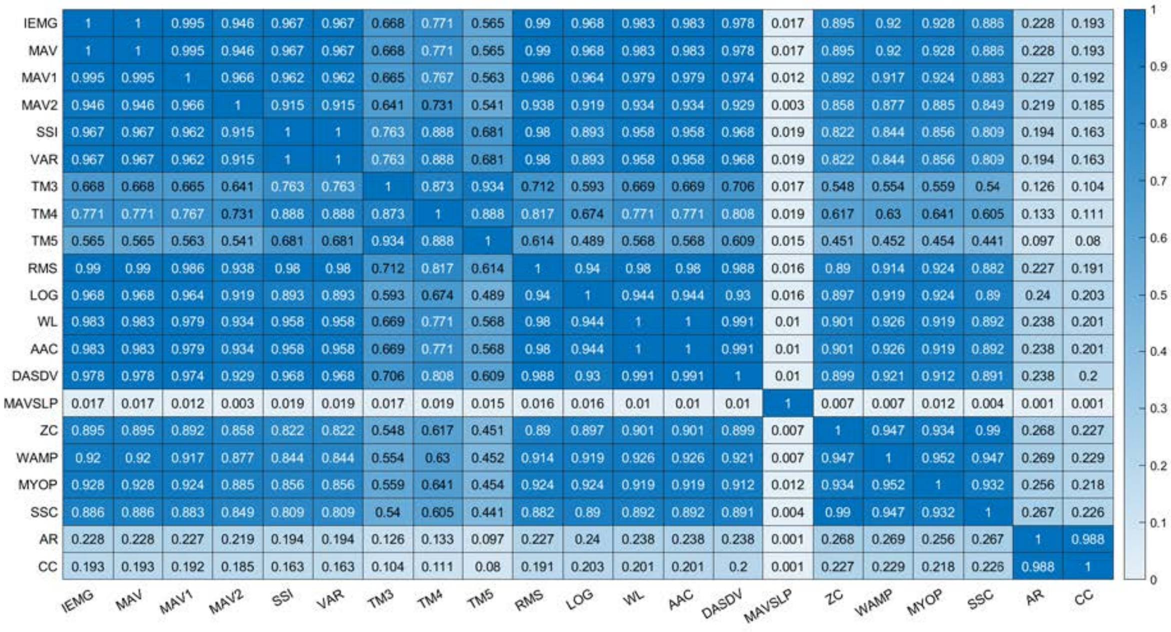

Figure S24. Confusion matrix of inter-feature PCC in Group 7.

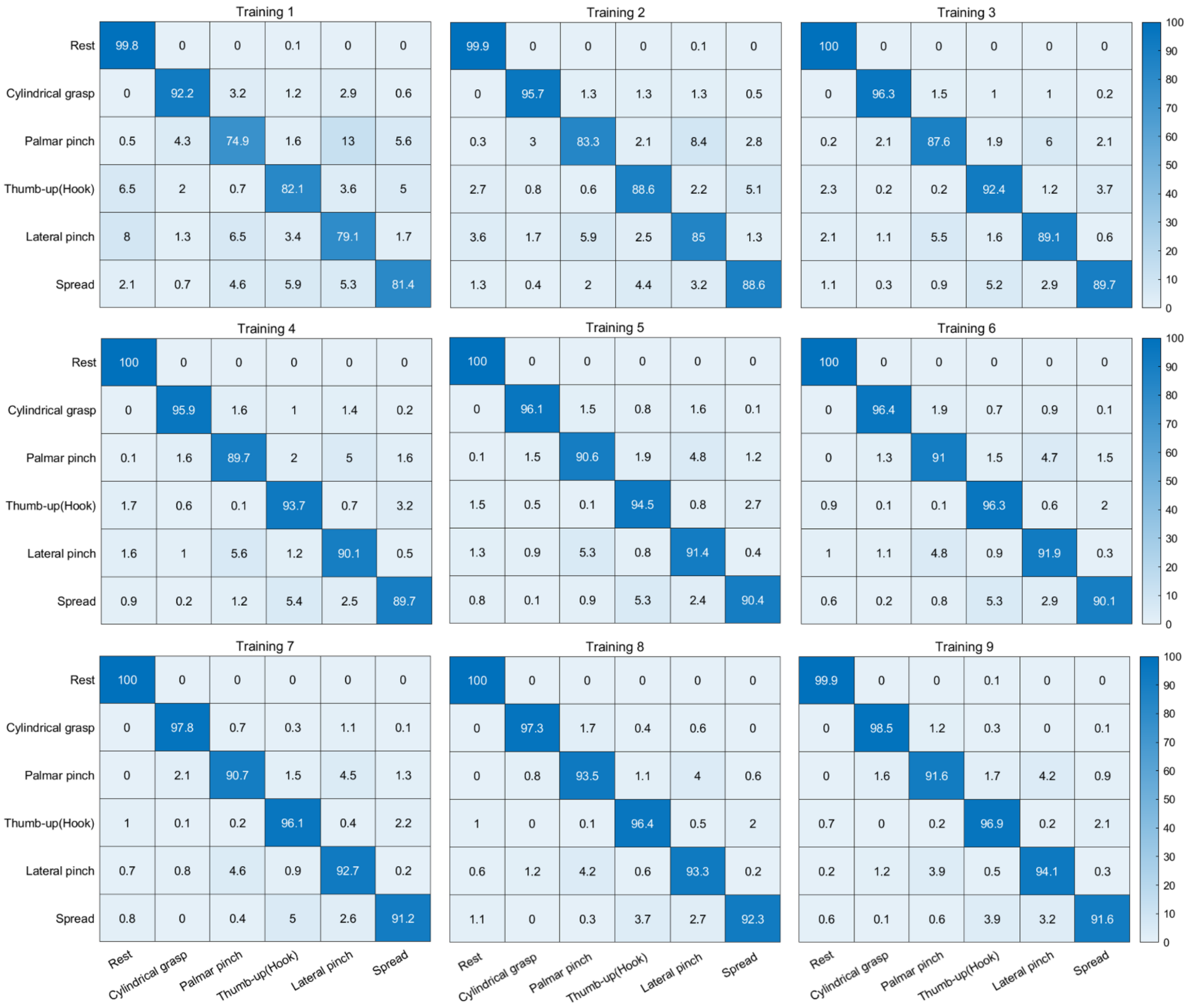

Figure S25. Confusion matrix of the hand postures of Group 1.

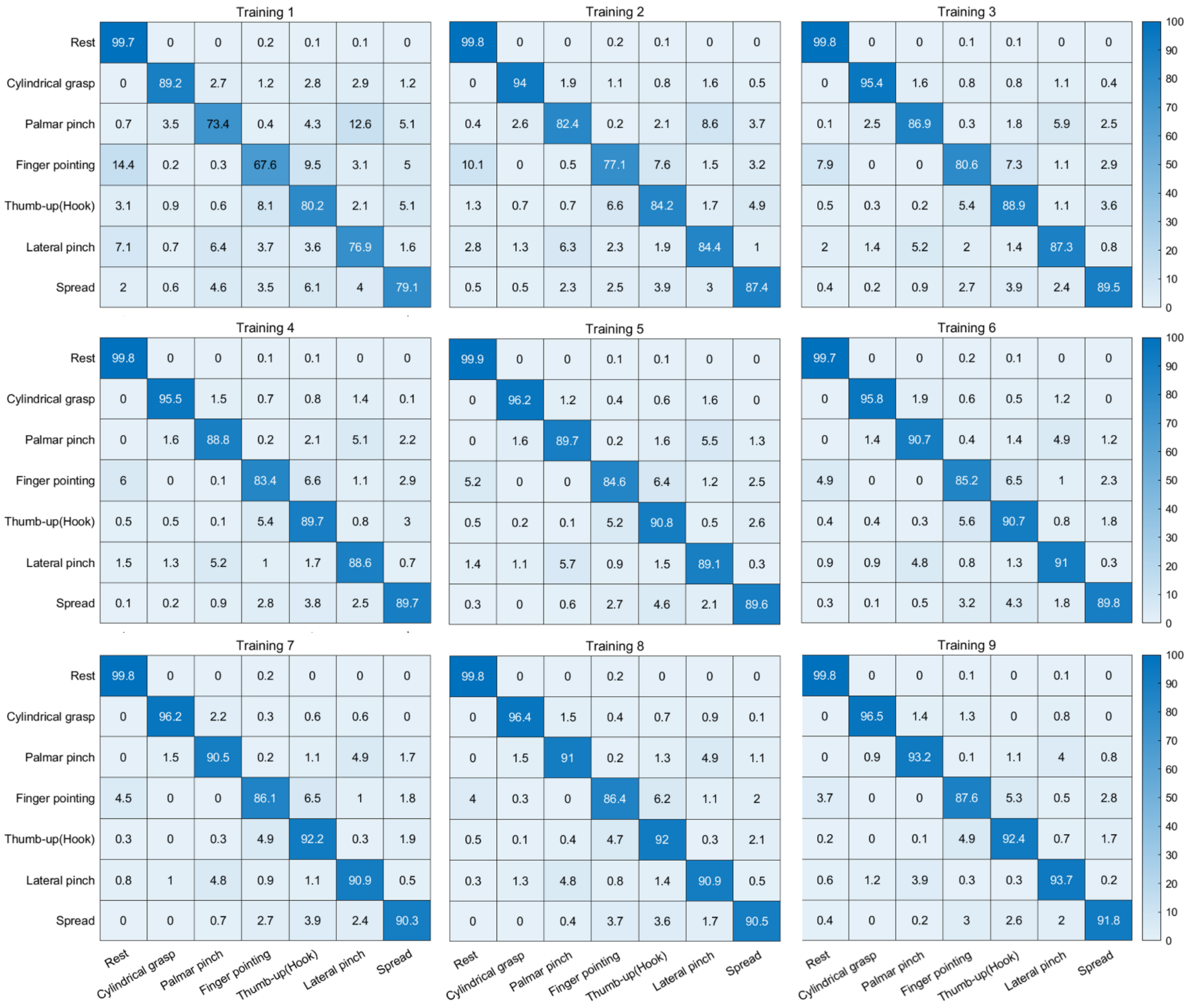

Figure S26. Confusion matrix of the hand postures of Group 2.

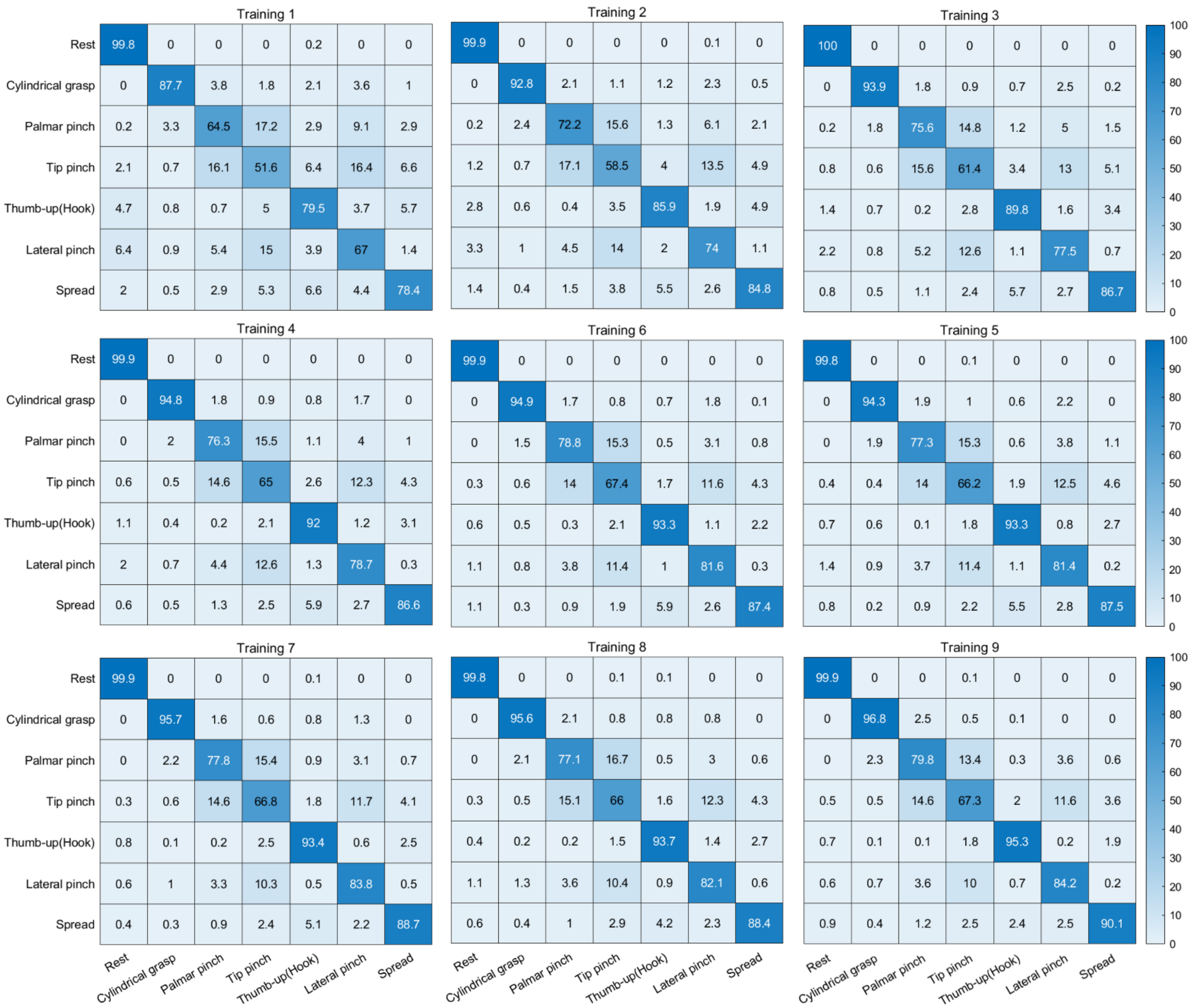

Figure S27. Confusion matrix of the hand postures of Group 3.

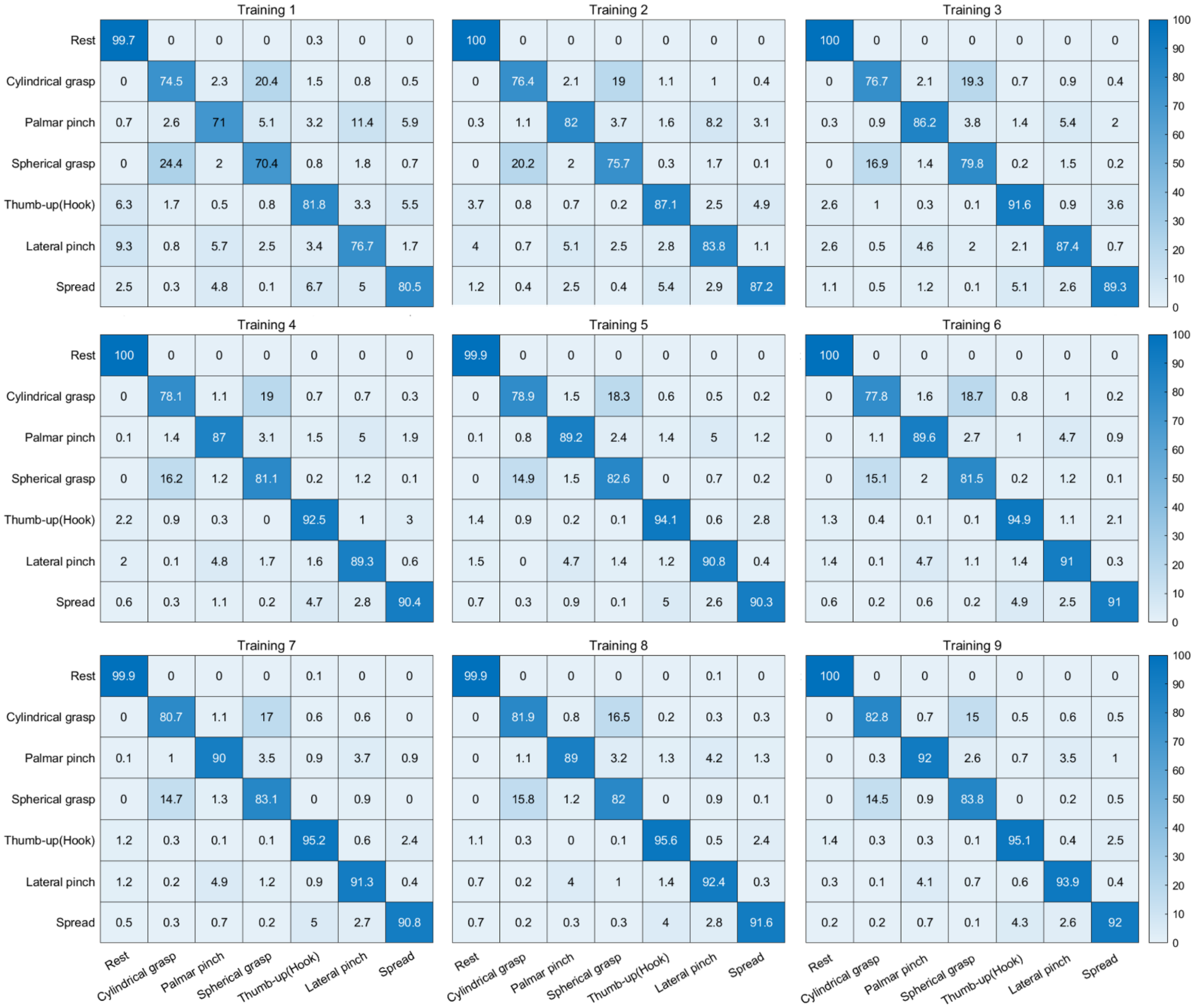

Figure S28. Confusion matrix of the hand postures of Group 4.

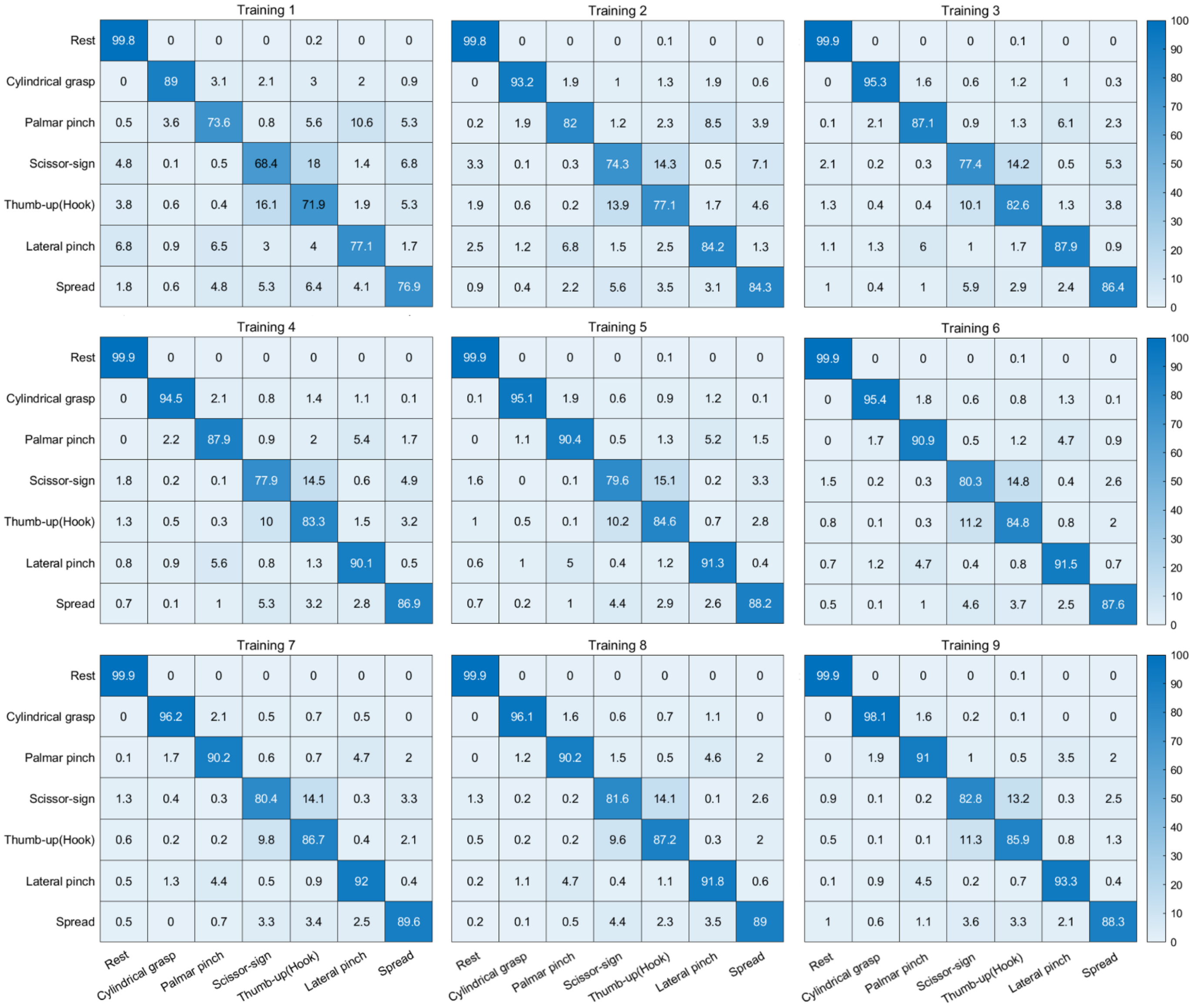

Figure S29. Confusion matrix of the hand postures of Group 5.

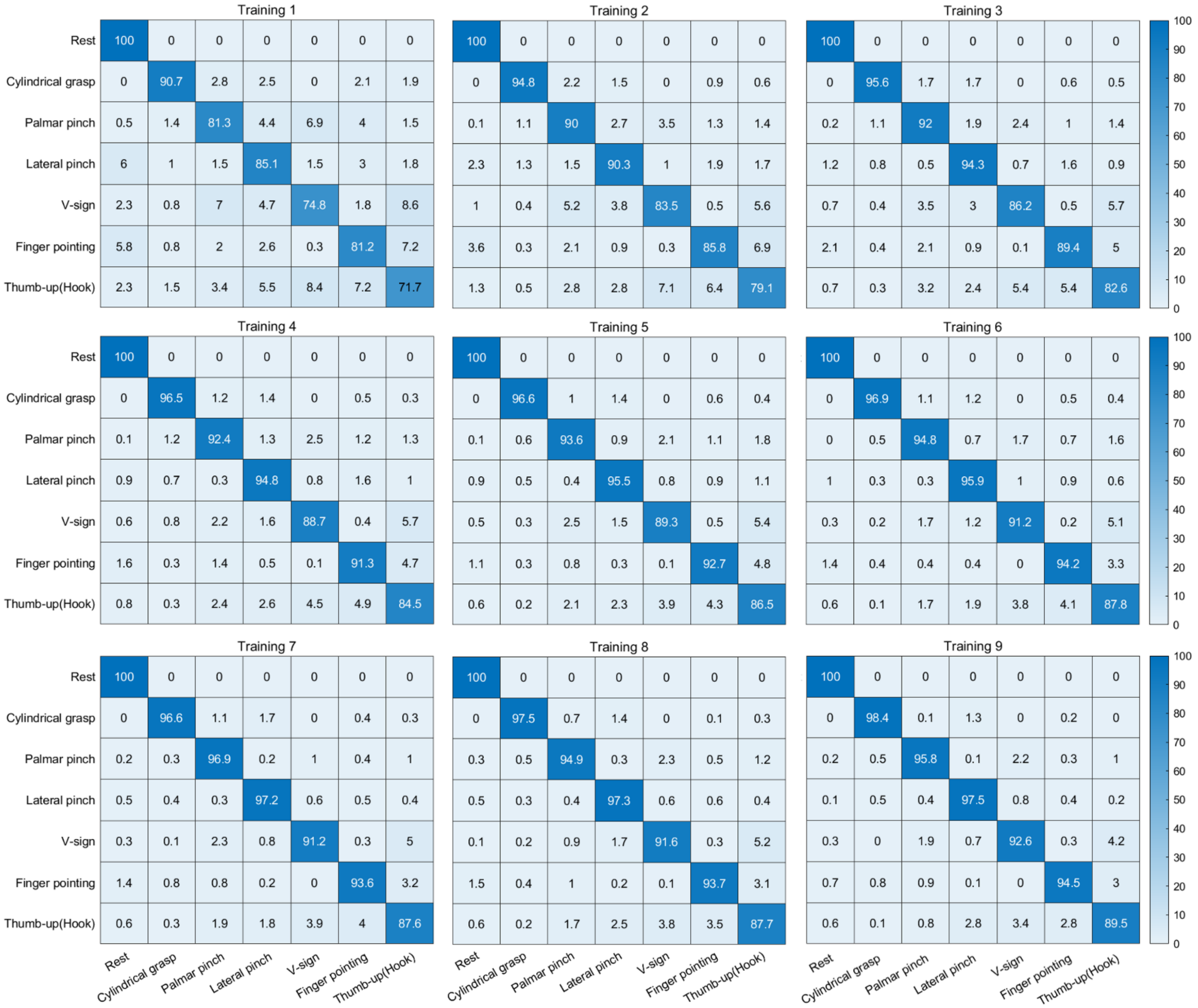

Figure S30. Confusion matrix of the hand postures of Group 6.

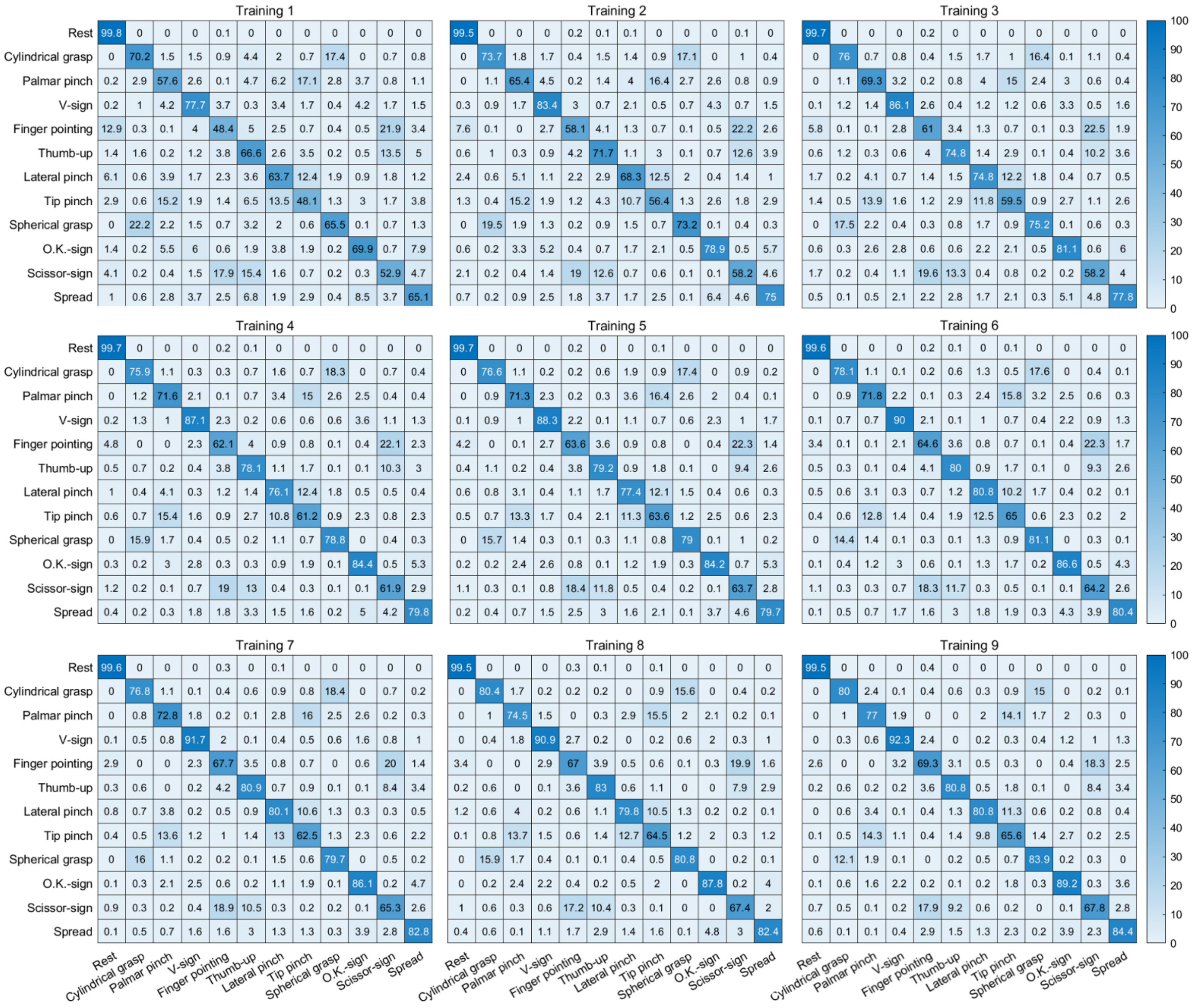

Figure S31. Confusion matrix of the hand postures of Group 7.
